# Mouse serum albumin induces neuronal apoptosis and tauopathies

**DOI:** 10.1186/s40478-024-01771-6

**Published:** 2024-04-23

**Authors:** Sheng-jie Hou, Ya-ru Huang, Jie Zhu, Ying-bo Jia, Xiao-yun Niu, Jin-ju Yang, Xiao-lin Yu, Xiao-yu Du, Shi-yu Liang, Fang Cui, Ling-jie Li, Chen Tian, Rui-tian Liu

**Affiliations:** 1 National Key Laboratory of Biochemical Engineering, Institute of Process Engineering, Chinese Academy of Sciences, Haidian District, Beijing, 100190 China; 2 University of Chinese Academy of Sciences, Beijing, 100049 China; 3 Ningxia University, Yinchuan, 750021 Ningxia China

**Keywords:** Mouse serum albumin, Neuroinflammation, Apoptosis, Tau phosphorylation

## Abstract

The elderly frequently present impaired blood–brain barrier which is closely associated with various neurodegenerative diseases. However, how the albumin, the most abundant protein in the plasma, leaking through the disrupted BBB, contributes to the neuropathology remains poorly understood. We here demonstrated that mouse serum albumin-activated microglia induced astrocytes to A1 phenotype to remarkably increase levels of Elovl1, an astrocytic synthase for very long-chain saturated fatty acids, significantly promoting VLSFAs secretion and causing neuronal lippoapoptosis through endoplasmic reticulum stress response pathway. Moreover, MSA-activated microglia triggered remarkable tau phosphorylation at multiple sites through NLRP3 inflammasome pathway. Intracerebroventricular injection of MSA into the brains of C57BL/6J mice to a similar concentration as in patient brains induced neuronal apoptosis, neuroinflammation, increased tau phosphorylation, and decreased the spatial learning and memory abilities, while Elovl1 knockdown significantly prevented the deleterious effect of MSA. Overall, our study here revealed that MSA induced tau phosphorylation and neuron apoptosis based on MSA-activated microglia and astrocytes, respectively, showing the critical roles of MSA in initiating the occurrence of tauopathies and cognitive decline, and providing potential therapeutic targets for MSA-induced neuropathology in multiple neurodegenerative disorders.

**Supplementary Information:**

The online version contains supplementary material available at 10.1186/s40478-024-01771-6.

## Introduction

Increasing evidence indicated that the occurrence and development of neurodegenerative diseases are closely associated with loosed or destructed blood–brain barrier (BBB) which are frequently present in the elderly [37]. BBB plays a critical role in stabilizing the internal environment of the central nervous system (CNS) by tightly controlling the entry of substances, including plasma proteins, drugs and harmful substances [31], while destructed BBB allows much more plasma components to enter the brain parenchyma, initiating cascading neuropathology [32]. One of the main plasma components leaking through destructed BBB is albumin, which is the most abundant protein in the plasma, and plays a vital role in transporting metabolites, nutrition, and maintaining colloid osmotic pressure. BBB disruption increases albumin levels in the cerebrospinal fluid (CSF) and the brains of elderly and the patients with Alzheimer’s disease (AD), frontotemporal dementia (FTD) or other neurodegenerative disease patients [18, 36]. After entering brain tissue, albumin can activate the microglia, and promote neuroinflammation [13], and induce excitatory synaptogenesis through astrocytic TGF-β/ALK5 signal, thereby promoting epilepsy occurrence [2]. However, the association of leaked albumin with neurodegenerative pathogenesis remains largely unknown.

Tau belongs to the family of microtubule-associated proteins, which maintains polymerization and stabilization of microtubules in neuronal axons and dendrites for ensuring essential cargo transportation [38]. Tau can be physiologically phosphorylated at Ser202/Thr205, Thr181, Thr217, Thr231, Ser396, Ser404, and Ser416 by multiple kinases and phosphatases, such as glycogen synthase kinase-3β (GSK-3β) [35] and calcium/calmodulin activated protein kinase IIα (CaMKII) [40]. However, under a pathological state, more tau becomes hyperphosphorylated, aggregates into oligomers and neurofibrillary tangles, causing synaptic impairment [7] and neuronal loss, and finally resulting in neuronal degeneration and cognitive decline [29]. Hyperphosphorylation of tau is one of the key pathological hallmarks in several neurodegenerative diseases, such as AD, FTD, Progressive supranuclear palsy, Cortical basal degeneration and other tauopathies [5]. However, the annotations on the regulation of tau hyperphosphorylation remains far from complete.

Very long-chain saturated fatty acids (VLSFAs) are saturated fatty acids with 20 or more carbons, primarily synthesized through elongation of very-long-chain fatty acids (Elovl) enzymes [17]. VLSFAs exert a variety of cellular functions and are associated with numerous diseases including cardiovascular disease, diabetes and neurodegenerative disorders [3, 20]. VLSFAs levels can also act as predictive biomarkers of AD [8]. In the CNS, A1 astrocytes release VLSFAs that induce neuronal apoptosis and finally lead to memory loss [12].

In this study, we investigated the effect of MSA on the cell phenotypes and function of microglia, astrocytes and neurons in vitro and in vivo, detected the cellular logic orders by which MSA influenced CNS, explored the association of MSA with tau phosphorylation, neuronal apoptosis, neuroinflammation and mouse cognition, and the corresponding underlying mechanisms.

## Materials and methods

### Animals

2-month-old C57BL/6J female littermates were purchased from Beijing HFK Bioscience Co.,Ltd. (Beijing, China). All mice were kept in the animal facility of Tsinghua University, and kept in a colony room at 22 ± 2 °C temperature and 45% ± 10% humidity under a 12 h:12 h light/dark cycle. In this study, all animal experiments were performed in accordance with the China Public Health Service Guide for the Care and Use of Laboratory Animals. Experiments involving mice and protocols were approved by the Institutional Animal Care and Use Committee of Tsinghua University.

### Primary microglia and astrocytes cultures

Microglia and astrocyte were purified by immmunopanning from postnatal day 5 mice and cultured as previously described [4, 10]. Briefly, micro-dissected cortical tissue was digested for 10 min at 37 °C in papain solution (Worthington Biochemical, LS003126), then gently disassociated with a 15 mL pipette to obtain a single-cell suspension. Homogenized cell suspensions were filtered through a 40-micron strainer (BD Bioscience) and the flow-through was centrifuged at 1500 rpm for 10 min to pellet cells. DMEM/F-12 medium (Sigma, D2906) was added to the pellet to resuspend the cells, and cells were placed for 40 min in a 37 °C incubator. The cell suspensions were allowed the CD11b antibodies (ThermoFisher Scientific, 14-0112-82) coated-immunopanning dish for 20 min at room temperature. Unbound cells and debris were removed by washing the dish 10 consecutive times with PBS. Isolated microglia were cultured in a defined serum-free medium containing 49 mL of phenol red free DMEM/F-12 medium containing 500 uL Penicillin–Streptomycin (ThermoFisher Scientific,15,140,122), 2 mM L-glutamine (ThermoFisher Scientific, 25,030,081), 5 μg/mL N-acetyl cysteine (Sigma, A8199), 5 μg/mL insulin (Merck, I9278), 100 μg/mL transferrin (Sigma, T8158), and 100 ng/mL sodium selenite (ThermoFisher Scientific, 11,360,070). Perform a 50% media change every 3 days to maintain the cultures. Cells cultured for 2 weeks were used for the following experiments.

After first immunopanning away microglia, the cell suspensions were added to the O4 antibodies (Sigma, O7139) coated-immunopanning dish for 15 min to remove oligodendrocytes. ITGB5 antibodies (ThermoFisher Scientific,14–0497-82) coated-petri plates was used to isolate astrocytes from remaining cells in suspension. After 40 min of incubation at room temperature, unbound cells and debris were removed by washing the dish 10 consecutive times with PBS. Isolated astrocytes were cultured with a defined serum-free base media including 50% neurobasal, 50% DMEM/F-12, 500 uL Penicillin–Streptomycin, 1mM sodium pyruvate (ThermoFisher Scientific, 11,360,070), 292 μg/mL L-glutamine, 1 μg/mL transferrin, 0.16 μg/mL putrescine (Sigma, P5780), 1 nM progesterone (Merck, P0130), 0.4 ng/mL sodium selenite, 5 ng/ml HBEGF (MedChemExpress, HY-P7400) and 5 μg/ml of N-acetyl cysteine. Perform a 50% media change every 7 days to maintain the cultures. Cells cultured for 2 weeks were used for the following experiments.

### Primary neurons cultures

The primary neurons were isolated from cortex and hippocampus of fetal mice as previously described [33]. Briefly, fetal mice were dissected to isolate the cortical and hippocampal tissue, and carefully peel off the meninges and blood vessels. The fresh brain tissue was cut into small pieces and the minced tissue was incubated in papain at 37 °C for 10 min. After the cells were gently blown into single cells using a pipette, the suspension was filtered with a 40-micron strainer and centrifuged at 1000 rpm for 5 min. Neurons were plated in DMEM medium containing 10% fetal bovine serum (FBS). DMEM medium was replaced with Neurobasal supplemented with B27 (ThermoFisher Scientific, 17,504,044), GluMax (ThermoFisher Scientific, 35,050,061), and pen-strep after 1 h of culture. The medium was replaced every 3 days, and primary neurons were cultured for 2 weeks to use.

### Conditional medium preparation

To explore the direct effect of MSA (CUSABIO, CSB-NP000801m) on microglia, astrocytes and neurons, MSA at 7 μM, a similar concentration in patients’ brains, was added to primary microglia, astrocytes and neurons respectively. 24 h later, cells were harvested and used for the detection via RT-qPCR, ICC and Elisa.

To explore the effects of MSA on neurons in a co-culture mode of microglia and astrocytes, we added MSA to co-cultures of mouse primary microglia and astrocytes. After 12 h, replaced culture medium with fresh medium and continue to culture the glia cells for 24 h. Glia medium (GM) was collected and cultured neurons for 48 h. Neurons were then used for subsequent experiments.

To elucidate the effect of MSA-activated microglia on astrocytes and neurons, we firstly added MSA to microglia. After 12 h, replaced culture medium with fresh microglia growth medium and continued to culture the microglia for 24 h. Microglia medium (MM) was collected and used to treat astrocytes and neurons for 24 h, respectively. Astrocytes and neurons were used for subsequent experiments 24 h after treated with MM.

To elucidate the effect of MSA-activated astrocytes on microglia and neurons, we added MSA to astrocytes. After 12 h, replaced culture medium with fresh astrocyte growth medium and continued to culture the astrocytes for 24 h. Astrocyte medium (AM) was collected and cultured microglia and neurons for 24 h, respectively. Microglia and neurons were used for subsequent experiments 24 h after treated with AM.

MAM preparation: MSA firstly stimulated microglia, after 12 h, the medium was replaced with fresh microglia growth medium. After 24 h, supernatant was collected and continued to culture astrocytes for 12 h. Then, the culture medium was replaced with fresh astrocyte growth medium and continued to culture for 24 h. Microglia-Astrocyte Medium (MAM) was collected and added to neurons for 48 h. Neurons were collected for subsequent experiments.

AMM preparation: MSA firstly stimulated astrocytes, after 12 h, the medium was replaced with fresh astrocyte growth medium. After 24 h, supernatant was collected and continued to culture microglia for 12 h. Then, the culture medium was replaced with fresh microglia growth medium and continue to culture for 24 h. Astrocyte-Microglia Medium (AMM) were collected and added to neurons for 48 h. Neurons were collected for subsequent experiments.

### Standard RT-qPCR

Gene expression of inflammatory factors, phenotypic markers, phagocytosis receptors and neurotrophic factors in glia and neurons were detected by RT-qPCR. Total RNA was extracted by using the RNeasy Lipid Tissue kit (Qiagen, #74,804) according to the manufacturer’s instructions. cDNA was prepared from total RNA using the PrimeScript RT-PCR kit (Takara, #RR037Q). Relative gene expression of the cDNA was assayed using a 7500 Fast real-time PCR instrument (Applied Biosystems) with SYBR Select Master Mix (Applied Biosystems, 4,472,908). qPCR data were analyzed by the ΔΔCT method by normalizing the expression of each gene to housekeeping gene β-Actin and then to the control groups. Primer sequences for mouse are listed in Additional file 1: Table S1.

### Neurotransmitter detection by biochemical kits

The levels of glutamic acid in neurons, both intracellularly and extracellularly, were detected with Glutamic Acid (Glu) Content Assay Kit (Solarbio, BC1585). Supernatant and cell were collected and processed for the detection of glutamate release follow the manufacturer’s protocol. Neuronal glutamic acid contents were determined by comparing the absorbance value with the calibration plot for standard solutions. The absorbance values were measured at 340 nm.

General Gamma-Aminobutyric Acid (GABA) ELISA Kit (Jonln, T0731) was used to measure GABA concentrations in neurons, both intracellularly and extracellularly. Supernatant and cells were collected and processed for the detection of GABA release follow the manufacturer’s protocol. Neuronal GABA contents were determined by comparing the absorbance value with the calibration plot for standard solutions. The absorbance values were measured at 645 nm.

### Western Blot (WB)

Total sample protein from cell and mice brain were extracted from RIPA protein lysate (Beyotime, P0013B) according to the manufacturer’s instructions. The concentration of protein was determined by the classical BCA protein determination method (Beyotime, P0010S). SurePAGE™ precast sodium dodecyl sulfate polyacrylamide gel electrophoresis (SDS-PAGE) gel (Mellun, MA0456) was used for electrophoresis. After electrophoresis, protein samples were transferred to PVDF membrane at 300 mA constant current for 0.5–2 h. The membrane was sealed at room temperature for 1 h with 5% nonfat milk. Primary antibody was applied overnight at 4 °C. In the next day, after washed thrice for 5 min each with TBST, the membranes were coped with secondary antibody for 1 h. Then membranes were washed thrice again and imaged in Amersham Imager system (GE Healthcare). The images were analyzed by ImageJ software. The primary antibodies used in this study were described in Additional file 1: Table S2.

### Targeted lipidomics

When conditional medium prepared, MAM was collected, quickly frozen in liquid nitrogen, stored in dry ice, and sent to the Beijing Bio-Tech Pack Technology Company Ltd. The samples were analyzed by liquid chromatography-mass spectrometry (LC–MS) analysis.

#### Immunohistochemistry (IHC)

Mice were anesthetized with chloral hydrate and transcardially perfused with cold PBS. The dissected mouse brains were fixed in 4% paraformaldehyde at 4°C overnight, dehydrated and embedded in paraffin. Making 5 μm paraffin sections, after dewaxing and hydration, antigen retrieval was carried out. After permeabilized with 0.3% Triton X-100 for 10 min, the sections were blocked in 10% donkey serum (Solarbio, SL050) for 1 h at room temperature. Subsequently, the sections were incubated with the primary antibodies overnight at 4 °C, and followed by corresponding fluorescently conjugated secondary antibodies, respectively, and imaged on a Leica TCS SP8 confocal microscope. For DAB staining, sections were incubated with appropriate secondary antibodies, and the staining was developed by incubating with DAB. Images of DAB-stained sections were captured using an Olympus BX61 microscope. The primary antibodies used in this study were described in Additional file 1: Table S2. All images were analyzed by ImageJ software.

#### Immunocytochemistry (ICC)

The cell slides in the 24-well plate were washed three times with PBS and fixed with 4% paraformaldehyde for 15 min. After permeabilized with 0.3% Triton X-100 for 10 min, the slides were blocked in 10% donkey serum for 1 h at room temperature. Subsequently, the sections were incubated with the primary antibodies overnight at 4 °C, and followed by corresponding fluorescently conjugated secondary antibodies, respectively, and imaged on a Leica TCS SP8 confocal microscope. The primary antibodies used in this study were described in Additional file 1: Table S2. All images were analyzed by ImageJ software.

#### Enzyme-linked immunosorbent assay

Levels of the inflammatory factors (IL-1β, TNF-α, IL-6) in samples from brain lysates of mice and cell culture supernatant were detected by ELISA kits (Neobioscience technology), according to the manufacturer’s protocols. The absorbance at 450 nm was measured using a SpectraMax M5 microplate reader.

Levels of Aβ in soluble or insoluble extractions of mouse hippocampal tissues were determined with Aβ 1–38 (Aβ38), Aβ 1–40 (Aβ40), and Aβ 1–42 (Aβ42) MSD Triplex assay kit (Meso Scale Discovery, Rockwilly, MA, USA, N45199A-1) according to the manufacturer’s instructions.

#### Stereotaxic injection

2-month-old C57BL/6J female mice were anesthetized with 1.2% tribromoethanol and placed in a stereotaxic device. The skull was exposed by a midline scalp incision, and a craniotomy was drilled unilaterally or bilaterally above each cannula implantation or injection site.

For the injection of MSA, the cannulas were placed into the lateral ventricles ( − 0.2 mm anteroposterior, 1.4 mm mediolateral, 1.8 mm dorsoventral). Insert the infusion needle attached to the 10 μL micro-syringe into the guide tube, 1 μL of MSA solution is delivered within 2 min. The injections were given every 4 day for 16 days (total of 5 injections) and every 4 day for 60 days (total of 16 injections) respectively.

In order to reduce the expression of Elovl1, adeno-associated virus (AAV) carrying Elovl1 shRNA was provided by OBiO Technology (Shanghai) Corp., Ltd. The sequence of Elovl1 shRNA was GAATCATGGCTAATCGGAAGC. The serotype of AAV was 2/5. For AAV injections, 3 μL (6.03 × 10^12^ vg/mL) of shElovl1 was injected bilaterally into the cortex (+ 1.3 mm anteroposterior, 0.7 mm mediolateral, 0.8 mm dorsoventral) and hippocampus ( − 2 mm anteroposterior, 1.7 mm mediolateral, 1.6 mm dorsoventral). Mice were injected with AAV one month before MSA treatment. After each injection, the needle was left in place for 2 min and then slowly withdrawn. The surgical site was cleaned with sterile saline and the incision sutured. After surgery, animals were monitored and provided post-surgical care.

#### Behavioral tests

Novel object recognition (NOR) test, Y-maze test and Morris water maze (MWM) test were applied to detect the memory and cognitive function of mice. NOR, a method for exploring animal recognition and memory of new objects, is based on the instinct of mice to explore the characteristics of new objects. In the adaptation phase, each mouse was allowed to freely explore the open-field area (a white box 40 cm wide × 40 cm deep × 40 cm high) during 5 min. In the training phase, mice were exposed to two identical objects, which they were allowed to freely explore for 5 min. Recognition memory was tested after 6 h by exposing the mice to one familiar and one novel object. The times that the mice explored the novel and the old object were recorded independently. The discrimination index was determined by performing the following calculation: (Time_novel_-Time_old_) / (Time_novel_ + Time_old_).

Y‐maze test was used to evaluate the spatial working memory of mice. The Y-maze test was performed in the Y-shaped maze having three arms. Three arms were randomly defined as novel arm, the starting arm and other arm. The arm with the dotted line represents the randomly chosen starting arm; the blocked off arm represents the novel arm. During the first training trial, each mouse was allowed to freely explore the starting arm and the other arm for 10 min. After a one-hour interval, the mice were allowed to freely explore all three arms for 5 min. A camera mounted above the maze automatically records the distance traveled, arm entries, and the time spent in each arm.

Mice were tested for their spatial learning and memory abilities using the MWM test. The water maze consisted of a water pool (122^2^ cm in diameter) containing opaque water and a platform (10^2^ cm in diameter) submerged 1 cm below the water surface. During learning trials for 5 days, mice freely swam for 60 s to find the hidden platform. Mice that failed to find the platform within 60 s were guided to and remained on the platform for 10 s. The mice were trained twice a day with a 4 h interval between training sessions. To evaluate spatial memory retention, the platform was removed for a probe trial 24 h after the last day of hidden platform training. The swimming path of the mouse was recorded by video camera and analyzed by Ethovision XT version 16.0 (Noldus software).

### Adult microglia isolation

Adult microglia were isolated from the mouse brain as previously described [1]. In brief, the brains of mice were minced in the Hibernate A (Thermo Fisher Scientific, A1247501)/B27 medium and dissociated for 15 min at 37 °C with 0.25% Trypsin–EDTA containing DNase I, after neutralized with DMEM supplemented with 10% FBS, cells were separated by Optiprep (Merck, D1556) density gradient centrifugation. Fractionated microglia from the mice treated with or without MSA were obtained for further RNA sequence analysis.

#### Adult astrocyte isolation

For adult astrocyte isolation, mice were perfused with cold PBS under isoflurane anesthesia, and then the brain was removed, dissected, and rinsed in HBSS. Enzymatic cell dissociation was then performed using an Adult Brain Dissociation Kit (Miltenyi Biotec, 130-107-677), according to the manufacturer’s instructions. The resulting single cell suspension was centrifuged at 300 g for 10 min at room temperature, resuspended in 40% Percoll and centrifuged at 800 g for 20 min with breaks off. After Fc receptors were blocked using anti-mouse CD16/CD32 (eBioscience, 14-0160-82) for 10 min, cells were incubated with ACSA-2-PE for 30 min on ice, and washed with 1 ml of blocking buffer and sorted using a FACSAria II cell sorter. Fractionated astrocytes from the mice treated with or without MSA were obtained for further RNA sequence analysis.

#### Adult neuron isolation

Adult neurons were isolated in an enriched population from the mice treated with or without MSA using the Mitlenyi MACS system, the Adult Brain Dissociation Kit (Miltenyi Biotec, 130-107-677), and Adult Neuron Isolation Kit (Miltenyi Biotec, 130-126-603). Mice were perfused with cold PBS under isoflurane anesthesia, and then the brain was removed, dissected, and rinsed in HBSS. The cortex and hippocampus tissues were isolated and transferred to a MACS C-tube. Samples was then dissociated using the appropriate pre-set protocol on the Miltenyi gentleMACS Octo Dissociator instrument with heaters attached. Fractionated neurons from the mice treated with or without MSA were obtained for further RNA sequence analysis.

#### RNA sequence and data analysis

The microglia, astrocytes and neurons isolated from the mice treated with or without MSA were sent to OE Biotech, Inc., (Shanghai, China) Company for RNA extraction and eukaryotic transcriptome sequencing. The RNA libraries were sequenced by OE Biotech, Inc., Shanghai, China. We are grateful to OE Biotech, Inc., (Shanghai, China) for assisting in sequencing and/or bioinformatics analysis. Bioinformatic analysis was performed using the OECloud tools at https://cloud.oebiotech.com/task/. The volcano map (or other graphics) was drawn based on the R (https://www.r-project.org/) on the OECloud platform (https://cloud.oebiotech.com/task/).

### Statistical analysis

Data were analyzed with GraphPad Prism v.9.5.1. Each figure legend denotes the statistical analysis used. All data are represented as mean ± s.e.m. For comparisons between groups, first, it was determined whether the data were normally distributed using the Shapiro–Wilk test (Sigma-Plot). If data were normally distributed, one-way ANOVA was used with post hoc Holm–Sidak test for pairwise comparisons or an unpaired *t*-test with two-tailed *p* values. If not, Mann–Whitney rank sum test (two groups) or Kruskal–Wallis one-way ANOVA on ranks (three or more groups) with post hoc Dunn’s test was used. In all cases, statistical difference was considered significant at *p < 0.05; **p < 0.01; ***p < 0.001; ****p < 0.0001.

## Results

### MSA directly activates microglia and astrocytes but not neurons

In the brain, neurons, astrocytes, microglia live and interact in the same complex microenvironment, to clarify the impact of MSA on CNS, we first investigated the direct effect of MSA on CNS cells, respectively, then explored the indirect effect of MSA on CNS by various conditional medium (CM). Firstly, primary microglia, astrocytes and neurons were individually treated with MSA at 7 μM, a pathological concentration in patients’ brains [36]. MSA significantly reduced average branch length, and number of end-point voxels and branches of microglia (Additional file 1: Fig. S1a, b), switched microglia from an "M0" phenotype with homeostatic molecular and functional signature having lower transcription levels of P2ry12, Tmem119, Tgfbr1, Smad3, Entdp1 and higher levels of Clec7a, Lgals3, Gpnmb, SPP1, Itgax to a microglia neurodegenerative phenotype (MGnD) (Additional file 1: Fig. S1c), promoted the production of proinflammatory factors such as TNF-α, IL-1α, IL-1β, IL-6 and C1q (Additional file 1: Fig. S1d), and inhibited the transcription of microglial phagocytosis-related receptor such as CD36, TLR4 and TREM2 (Additional file 1: Fig. S1e).

MSA also changed astrocyte morphology by significantly reducing their number of end-point voxels and branches but not their average branch length (Additional file 1: Fig. S1f, g), induced astrocytes to reactive A1 phenotype with increased transcription levels of H2-T23, Serping-1, H2-D1, Ggta-1, and decreased levels of S100a10, Cd109 (Additional file 1: Fig. S1h), enhanced levels of proinflammatory factors such as IL-6, CCL2, GM-CSF (Additional file 1: Fig. S1i), and reduced levels of neurotrophic factors, including Thbs2 and Sparcl1 (Additional file 1: Fig. S1j). However, MSA did not show apparent impact on neuron morphology (Additional file 1: Fig. S2a, b). It also did not change the intracellular and extracellular levels of excitatory (Glutamic acid) and inhibitory (GABA) neurotransmitters (Additional file 1: Fig. S2c, d), and the levels of glutamate transporter1 (vGLUT1), GABA transporter1 (vGAT) (Additional file 1: Fig. S2e, f, g). These data indicated that MSA activated microglia and astrocytes to inflammatory states, but did not exhibit apparent effect on neuron morphology and function.

### AM activates microglia and induces neuronal excitability

We next tested the effect of AM (conditional medium from MSA-treated astrocyte culture) on the microglia and neurons. we added AM to the culture of microglia and neurons, respectively, and then cultured for 48 h. AM treatment changed microglial morphology, and significantly reduced the microglial number of branches, average branch length and number of end-point voxels (Additional file 1: Fig. S3b, c). AM switched microglia to MGnD phenotype (Additional file 1: Fig. S3d) with enhanced transcription levels of inflammatory factors such as TNF-α, IL-1α, IL-1β, IL-6 (Additional file 1: Fig. S3e) and decreased transcription levels of phagocytosis-related genes (Additional file 1: Fig. S3f).

**Fig. 1:**
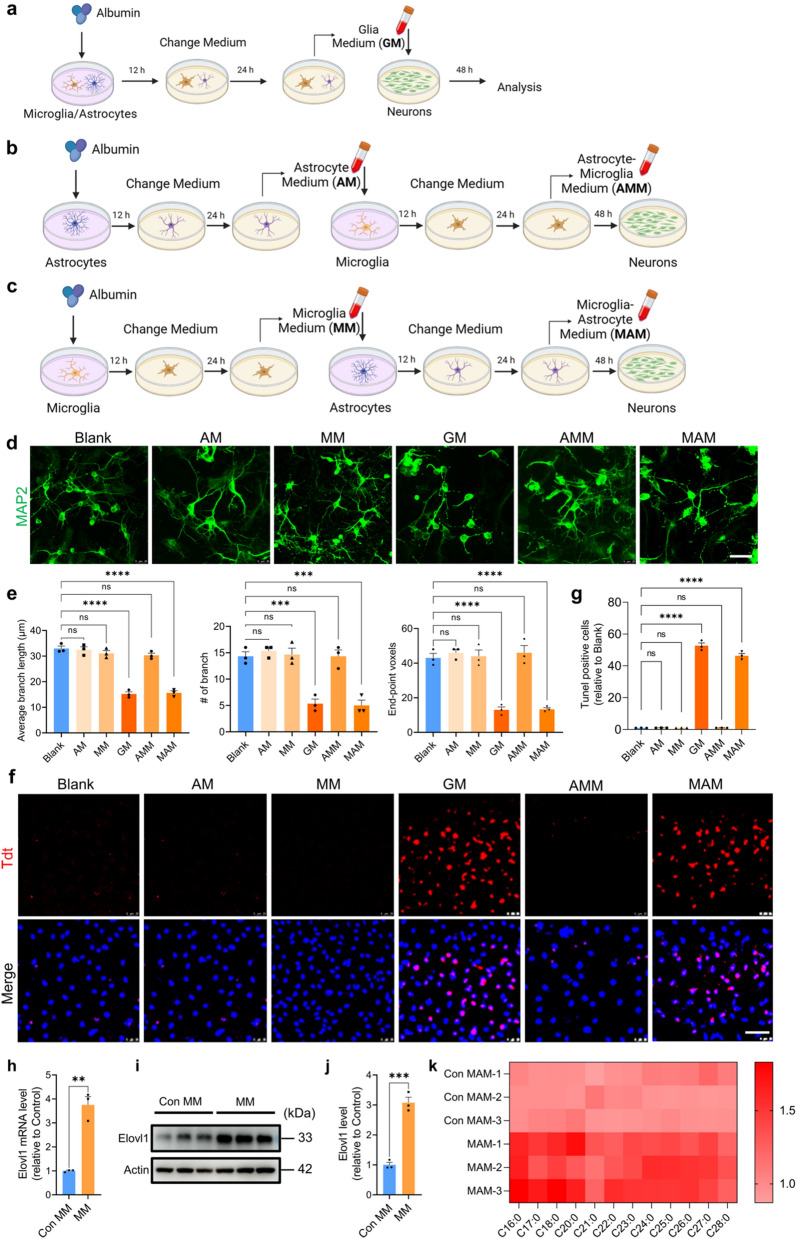
Astrocytic VLSFAs induced by MM leads to neuron lipoapoptosis. **a–c,** A schematic diagram describing the preparation of GM **a** AM, AMM **b** MM, MAM **c** and the neurons treated with various conditional medium. **d**, Representative confocal images of primary neurons treated by various conditional medium (scale bar, 25 μm). **e**, Statistical analysis of neurons morphology including average branch length, the number of end-point voxels and branch in **d** by Image J. **f**, Representative confocal images of neuronal Tunel^+^ nuclei in red following the treatment with various conditional medium (scale bar, 25 μm). **g**, The quantitation analysis of Tunel positive cells in **f** via Image J. For **e**, **g**, n = 3, representing three independent experiments, data are mean ± SEM. One-way ANOVA with Tukey’s multiple comparisons test for multiple groups was used for statistical analysis. **h**, The mRNA level of Elovl1 in astrocytes treated by MM determined by qPCR. **i**, The level of Elovl1 in MM-treated astrocytes detected by WB. **j**, Quantification of the WB in **i** by Image J. For **h**, **j**, n = 3, representing three independent experiments, data are mean ± SEM, and Student’s test was used for statistical analysis. **k**, Heat map showing the levels of saturated, long-chain FFAs in MAM. ns, no significance; *p < 0.05, **p < 0.01, ***p < 0.001 and ****p < 0.0001

Our Tunel staining showed that AM did not induce neuron apoptosis after 48 h of treatment (Fig. 1f, g), but it greatly raised the intracellular and extracellular levels of glutamate acid in neuron culture (Additional file 1: Fig. S3g, h), which then contributed to the rise in vGLUT1 (Additional file 1: Fig. S3i, k). Besides, AM did not affect the level of GABA and vGAT (Additional file 1: Fig. S3g– k). These results are consistent with the previous report that inflammatory factors in AM regulated neuronal excitability [43].

### MM activates astrocytes and induces neuronal excitability

We examined the effect of MM (conditional medium from MSA-treated microglia culture) on the astrocytes and neurons by adding MM to the culture of astrocytes and neurons, respectively (Additional file 1: Fig. S4a). MM significantly decreased the number of astrocytic branches and end-point voxels, but not average branch length (Additional file 1: Fig. S4b, c), and switched astrocytes to reactive A1 phenotype (Fig. S4d) with enhanced release of inflammatory factors such as IL-6, CCL2, GM-CSF (Fig. S4e), and almost complete loss of neurotrophic function (Fig. S4f).

Moreover, we found that MM induced an increase in neuronal excitatory neurotransmitters (Fig. S4g, h) and vGLUT1 (Additional file 1: Fig. S4i, j), but not in the levels of GABA and vGAT, and neuronal apoptosis (Fig. 1f, g). These results suggested that MM activated astrocytes and induced neuronal excitability.

### MAM but not AMM induces neuronal apoptosis

Previous researches have shown that neuron-glial interactions play an important role in neurodevelopment [19], synapse elimination [41] and neuron dysfunction and death [39]. In order to compare the direct and indirect effect of MSA-activated microglia or astrocytes on neuronal structure, function and survival, we prepared conditional medium (CM) including GM (Fig. 1a), AMM (Fig. 1b) and MAM (Fig. 1c) according to the protocol. The primary neurons were treated with various CM and the neuronal morphology and apoptosis were then examined. Interestingly, GM and MAM rather than MM, AM and AMM significantly decreased the number of neuronal branches and end-point voxels, and the average branch length (Fig. 1d, e), and induced neuron apoptosis detected by Tunel staining (Fig. 1f, g). These results indicated that the detrimental effect induced by MSA on neurons requires the sequential participation of microglia and astrocyte following a logic order.

### VLSFAs in MAM causes neuronal lipoapoptosis.

Previous reports have shown that VLSFAs were synthesized by metabolic enzyme Elovl1 and secreted by A1 astrocytes, induced neuronal apoptosis [12, 34].We here found that both mRNA and protein levels of Elovl1 in astrocytes were markedly upregulated with the treatment of MM (Fig. 1h, i, j). Correspondingly, the levels of VLSFAs such as C16:0 and C18:0 in MAM were significantly increased (Fig. 1k). To further verify this result, we treated astrocytes with Elovl1.

shRNA in the presence of MM (Additional file 1: Fig. S5a, b, c, d), and observed that the decreased Elovl1.

expression significantly prevented VLSFAs production (Additional file 1: Fig. S5p) and MAM induced the.

changes in neuronal morphology and apoptosis (Additional file 1: Fig. S5e–j). These results demonstrated that the CM from MSA-activated microglia increased Elovl1 levels in astrocytes, and promoted VLSFAs secretion, resulting in neuronal apoptosis.

Nonadipose tissues exposed to an excess of long-chain saturated fatty acids would occur lipoapoptosis [30]. In lipoapoptosis, saturated lipids activate the PERK (protein kinase R-like endoplasmic reticulum kinase) endoplasmic reticulum stress response pathway, causing apoptosis. We here detected key lipoapoptosis pathway indicators in neurons treated with MAM by western blot. The level of PERK phosphorylation was significantly upregulated, leading to EIF2A phosphorylation, FOXO3A dephosphorylation, which further enhanced the downstream PUMA expression, Bax activation, caspase-3 cleavage and neuronal apoptosis (Additional file 1: Fig. S5k, l).

### Cytokines induce Elovl1 generation

It has been reported that the cytokines IL-1α, TNF-α, and C1q from activated microglia induce the transformation of astrocytes to A1 phenotype [23]. To explore the mechanism by which MSA-activated microglia induced Elovl1 expression in astrocytes, we neutralized IL-1α, TNF-α, and C1q in MM with corresponding antibodies and then added the resultant CM to astrocyte culture. qPCR results showed that the neutralization with one antibody did not affect Elovl1 expression, while simultaneously neutralizing any two cytokines partly decreased Elovl1 expression by approximately 30%, and only when all three cytokines were simultaneously neutralized, Elovl1 expression was remarkably decreased (Additional file 1: Fig. S5m). Moreover, neutralization of three cytokines significantly reduced Elovl1 protein levels (Additional file 1: Fig. S5n, o). Correspondingly,

simultaneous neutralization of IL-1α, TNF-α and C1q obviously decreased VLSFAs production (Additional file 1: Fig. S5p) and ameliorated neuronal apoptosis and changes in morphology induced by MAM (Additional file 1: Fig. S5e–j). These findings suggested that microglia-produced IL-1α, TNF-α and C1q almost equivalently contributed to Elovl1 expression, and the combination of any two factors efficiently induced Elovl1 expression.

### AAV-shElovl1 inhibit the expression of Elovl1 in mice

To assess the effect of the AAV-shElovl1 on Elovl1 levels, we used IHC to detect AAV-shElovl1 expression and measured the total protein level of Elovl1 in the mouse brains. The IHC results showed that AAV-shElovl1 obviously expressed in astrocytes (Additional file 1: Fig. S6a). Elovl1 expression significantly increased in the mice treated with MSA compared to the control group, while AAV-shElovl1 significantly decreased Elovl1 expression in the mice (Additional file 1: Fig. S6b, c). This conclusion was further confirmed by detecting Elovl1 protein levels using WB (Additional file 1: Fig. S6d, e).

### MSA impairs spatial memory and cognition in mice

To determine the effect of MSA on the neuronal apoptosis, neuroinflammation and spatial learning and memory abilities in mouse model, we infused MSA into the lateral cerebral ventricle of C57BL/6J mice every 4 days for 16 days (MSA-mice). The control group was injected with the same volume of sterile PBS (PBS-mice). Moreover, in order to clarify the role of Elovl1 during MSA treatment, we injected adeno-associated virus (AAV) carrying the Elovl1 shRNA, which was specifically expressed in astrocytes, into the cortex and hippocampus of one mouse group one month before MSA injection.

**Fig. 2:**
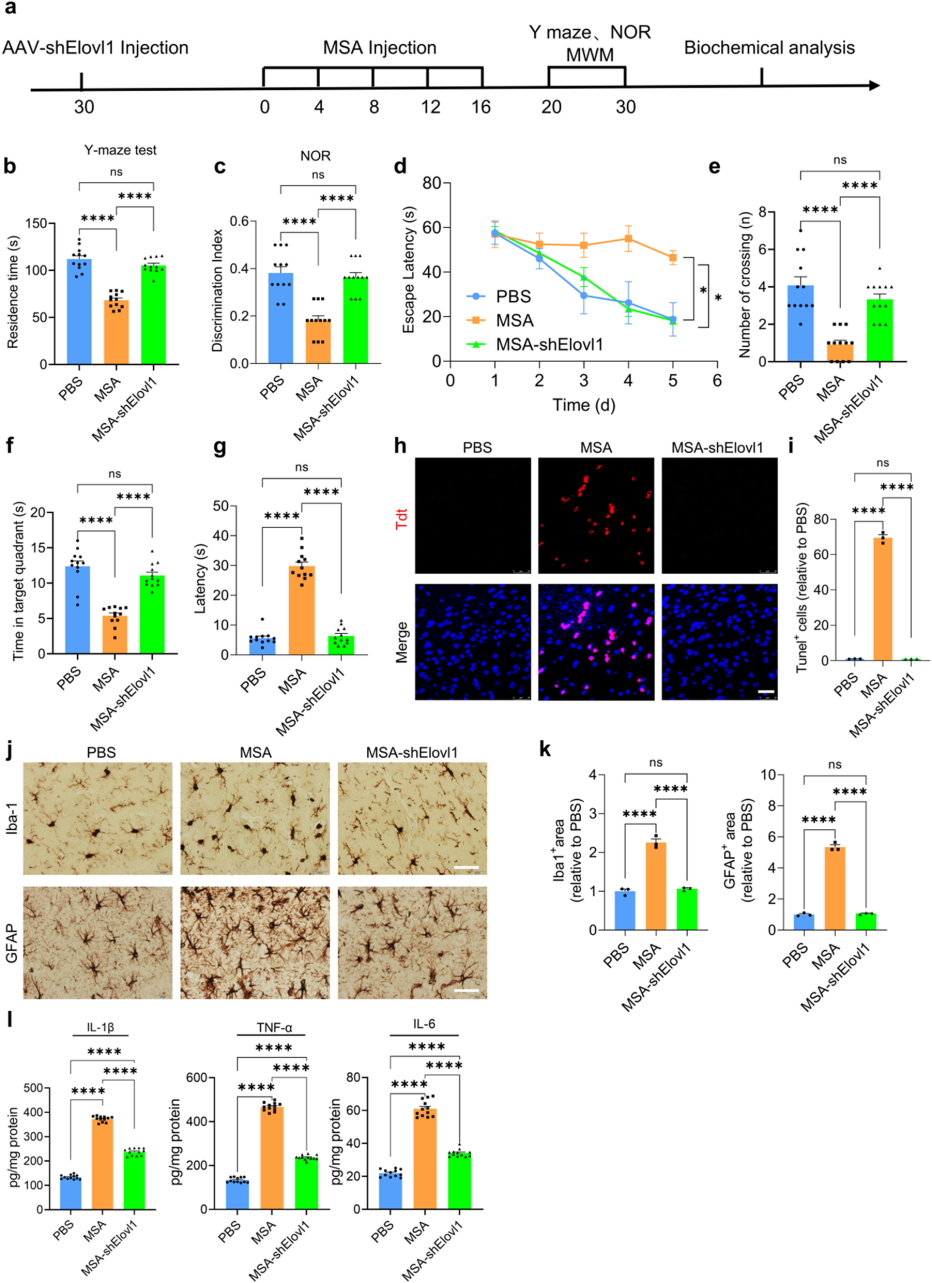
MSA impairs mouse learning and memory ability in mice. **a** Experimental procedure diagram in vivo**. b**, The time spent in the novel arm in Y-maze test for various groups. n = 12 mice per group. **c**, Discrimination index in the NOR test for various groups. n = 12 mice per group. **d,** The time for mice to reach the submerged hidden platform during training. **e**, The latency to find the position of the platform. **f**, The number of platform crossings. **g**, The time spent by the mice in the targeted quadrant. n = 12 mice per group. **h**, The apoptosis in mouse brains detected by Tunel staining (scale bar, 25 μm). **i**, The quantitation analysis of Tunel positive cells in **g**. **j**, Microgliosis and astrogliosis in mouse brains detected by IHC staining (scale bar, 100 μm). **k**, The quantification analysis of gliosis in **i** by Image J. **l**. Protein levels of inflammatory factors in mouse brains detected by corresponding Elisa kits. For **b–g**, **i**, **k**, **l**, data are mean ± SEM. One-way ANOVA with Tukey’s multiple comparisons test for multiple groups was used for statistical analysis. ns, no significance; *p < 0.05, **p < 0.01, ***p < 0.001and ****p < 0.0001

Y-maze and novel object recognition (NOR) tests were conducted to evaluate the spatial memory and short-term recognition memory of mice. The results of Y-maze test indicated that compared with PBS group, the time spent in the novel arm were significantly decreased in the MSA-mice group, while shElovl1 injection significantly improved the spatial memory with more time in the novel arm (Fig. 2a). NOR test showed the decreased recognition index for the novel object in MSA-mice, but the mice with Elovl1 knockdown exhibited a marked increase in discrimination index of the novel object (Fig. 2b). Consistently, our Morris water maze (MWM) test results indicated that the mice injected with MSA displayed significantly worsened spatial learning during the training phase by showing longer latency time than PBS group, while the group with Elovl1 knockdown obviously showed improved spatial learning ability with shorter latency time than MSA-mice (Fig. 2c). During the probe test with the platform being removed, the number of crossing platform position and the time in the target quadrant were more less, and the time to reach the position of platform was longer for MSA-mice relative to PBS-mice (Fig. 2d, e, f). However, the retention memory was remarkably improved in shElovl1-injected mice, as they crossed the platform location more times and spent longer time in the target quadrant than MSA-mice (Fig. 2c–f). These results suggested that MSA impaired mouse memory and recognition, and Elovl1 knockdown reversed the corresponding deficits in MSA-mice.

### MSA induces neuronal apoptosis and neuroinflammation in mice

Our Tunel staining showed that apparent apoptosis occurred in the hippocampi of MSA-mice, while shElovl1 treatment significantly decreased cell apoptosis (Fig. 2g, h). Moreover, MSA injection increased microgliosis and astrogliosis in mouse brains, while Elovl1 knockdown markedly attenuated the gliosis (Fig. 2i, j). Consistently, the levels of IL-1β, IL-6 and TNF-α were substantially enhanced in the brains of MSA-mice, while Elovl1 knockdown significantly decreased the levels of these inflammatory factors (Fig. 2k). These results implied that MSA induced neuronal apoptosis and neuroinflammation, and Elovl1 knockdown significantly reduced these detrimental effects induced by MSA.

### MSA induces tau phosphorylation in mice

**Fig. 3:**
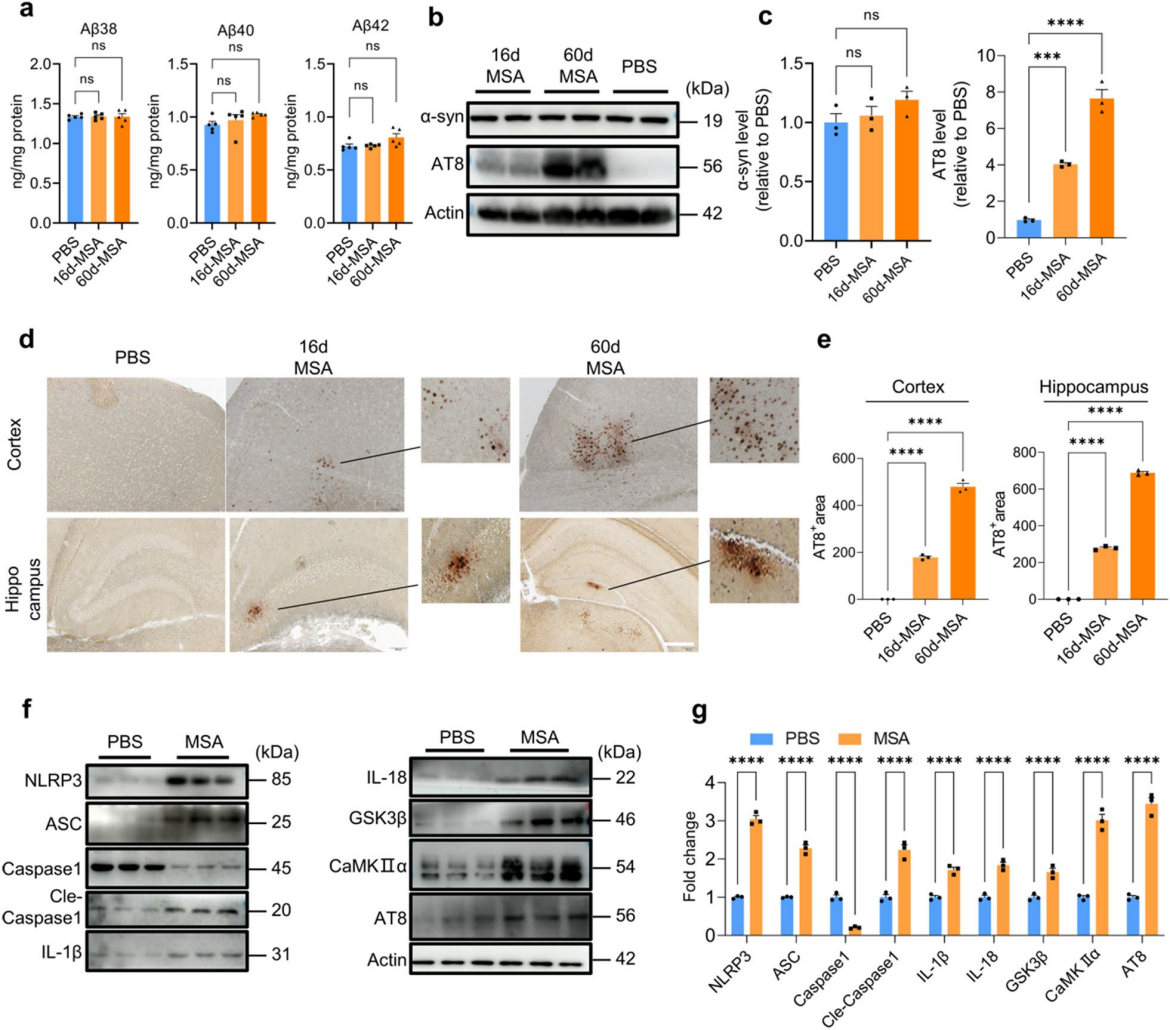
MSA induces tau phosphorylation in mouse brains. **a** Aβ levels in the brains of mice treated with or without MSA detected by MSD. n = 5, representing three independent experiments, data are mean ± SEM. One-way ANOVA with Tukey’s multiple comparisons test for multiple groups was used for statistical analysis. **b**, The levels of tau phosphorylation and α-synuclein in the mouse brains treated with or without MSA detected by WB. **c**, Quantification of the WB result in **b** by Image J. **d**, The tau phosphorylation in cortex and hippocampus of the mouse brains detected by IHC staining (scale bar, 200 μm). **e**, Quantification of AT8 staining in **d** by Image J. For **c**, **e**, n = 3, representing three independent experiments, data are mean ± SEM. One-way ANOVA with Tukey’s multiple comparisons test for multiple groups was used for statistical analysis. **f**, The levels of key proteins involved in the pathway of tau phosphorylation in mouse brains detected by WB. **g**, Quantitative analysis of the WB bands in **f** using Image J analysis software. n = 3, representing three independent experiments, data are mean ± SEM, and Student’s test was used for statistical analysis. ns, no significance; *p < 0.05, **p < 0.01, ***p < 0.001and ****p < 0.0001

Given MSA induced gliosis both in vitro and in vivo, we detected the effect of MSA on the amyloid generation as neuroinflammation may exacerbate amyloid pathology [21]. No significant changes were observed in the levels of Aβ38, Aβ40, Aβ42 and α-synuclein (α-syn) in the brain homogenates of MSA-mice compared to control mice, even though MSA was inject every 4 days for 60 days (Fig. 3a–c). However, the phosphorylation of tau at Ser202/Thr205 detected by WB was remarkably increased after MSA injection (Fig. 3b, c). Consistently, our IHC results showed that phosphorylated tau occurred both in the cerebral cortex and hippocampus of mice injected with MSA following 16 d and 60 d (Fig. 3d, e).

Activated NLRP3 inflammasome has been reported to drive microglia activation and tau pathology [16], and we here found that the levels of components of NLRP3 inflammasome, NLRP3, ASC and cle-caspase1 were significantly elevated in the brains of mice treated with MSA, suggesting NLRP3 inflammasome was activated (Fig. 3f, g). Enhanced cle-caspase1 led to IL-1β and IL-18 maturation, increasing their protein levels. IL-1β and IL-18 then upregulated the expression of GSK3β and CaMKIIα via their binding to IL-1 receptor on neurons, eventually promoting tau phosphorylation (Fig. 3f, g).

### MSA induces tau phosphorylation in a NLRP3-dependent manner

**Fig. 4:**
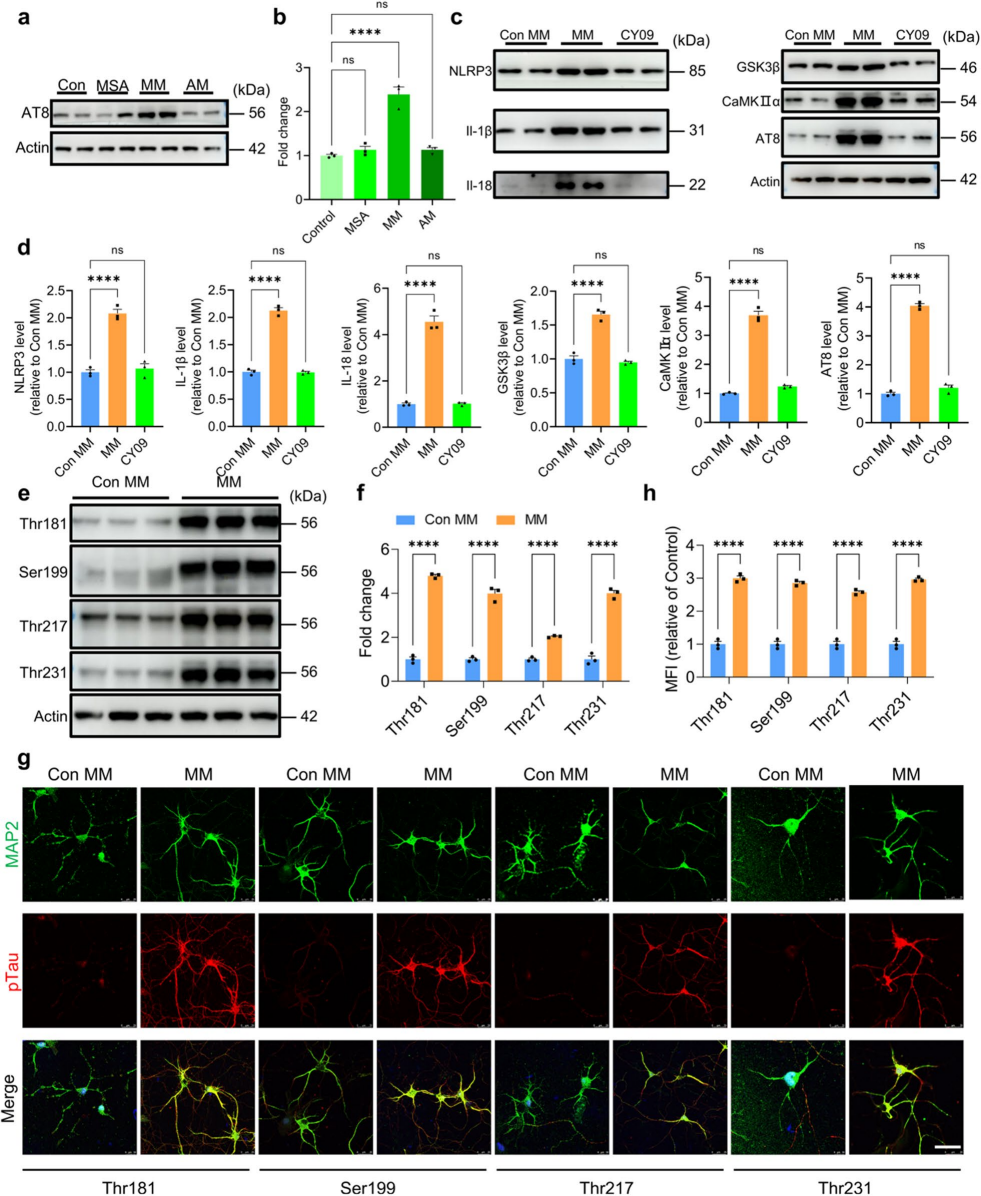
MSA-activated microglia induce tau phosphorylation in a NLRP3-dependent manner. **a** The levels of tau phosphorylation in primary neurons treated by MSA, MM and AM. **b** Quantification of the WB results in **a** via Image J. **c** The key protein levels in tau phosphorylation pathway in primary neurons in the presence or absence of CY09. **d** Quantitative analysis of the WB bands in **c** using Image J. For **b**, **d**, n = 3, representing three independent experiments, data are mean ± SEM. One-way ANOVA with Tukey’s multiple comparisons test for multiple groups in was used for statistical analysis. **e**, Phosphorylated Tau levels at multiple phosphorylation sites in neurons treated with or without MM. **f**, Quantitative analysis of the WB bands in (e) using Image J. **g**, Phosphorylated Tau levels at multiple sites in the primary neurons treated with or without MM detected by ICC (scale bar, 25 μm). **h**. Quantification of ICC results in (g) by Image J. For **f**, **h**, n = 3, representing three independent experiments, data are mean ± SEM, and Student’s test was used for statistical analysis for multiple groups was used for statistical analysis. ns, no significance; *p < 0.05, **p < 0.01, ***p < 0.001and ****p < 0.0001

To further investigate the mechanism of tau phosphorylation, MSA, MM and AM were used to treat primary neurons. Our WB results demonstrated that MSA did not directly induce tau phosphorylation, and MM rather than AM significantly caused tau phosphorylation, indicating that MSA-activated microglia induced tau phosphorylation (Fig. 4a, b). Consistent with the results in mouse brain, MM caused tau phosphorylation through a NLRP3-dependent manner, including the cleavage of caspase 1, maturation of IL-1b and IL-118, further upregulation of GSK3β and CaMKIIα, ultimately lead to the phosphorylation of tau. Furthermore, we added CY09, a specific inhibitor of NLRP3, into microglia culture, and found that CY09 treatment markedly reversed the effect of MM on tau phosphorylation in neurons by decreasing the levels of IL-1β and IL-18, and further reducing the levels of GSK3β, CaMKIIα and AT8 (Fig. 4c, d). These results indicated that MSA induced neuronal tau phosphorylation in a microglial NLRP3-dependent manner.

We next investigated the sites of tau phosphorylation induced by MSA. Western blot results displayed that the levels of tau phosphorylation at Thr181, Ser199, Thr217 and Thr231 were substantially elevated following MM treatment (Fig. 4e, f), which was further confirmed by ICC assay (Fig. 4g, h). These findings suggested that MSA-activated microglia induced neuronal tau phosphorylation at multiple sites.

### The effect of MSA on glia transcriptome

**Fig. 5:**
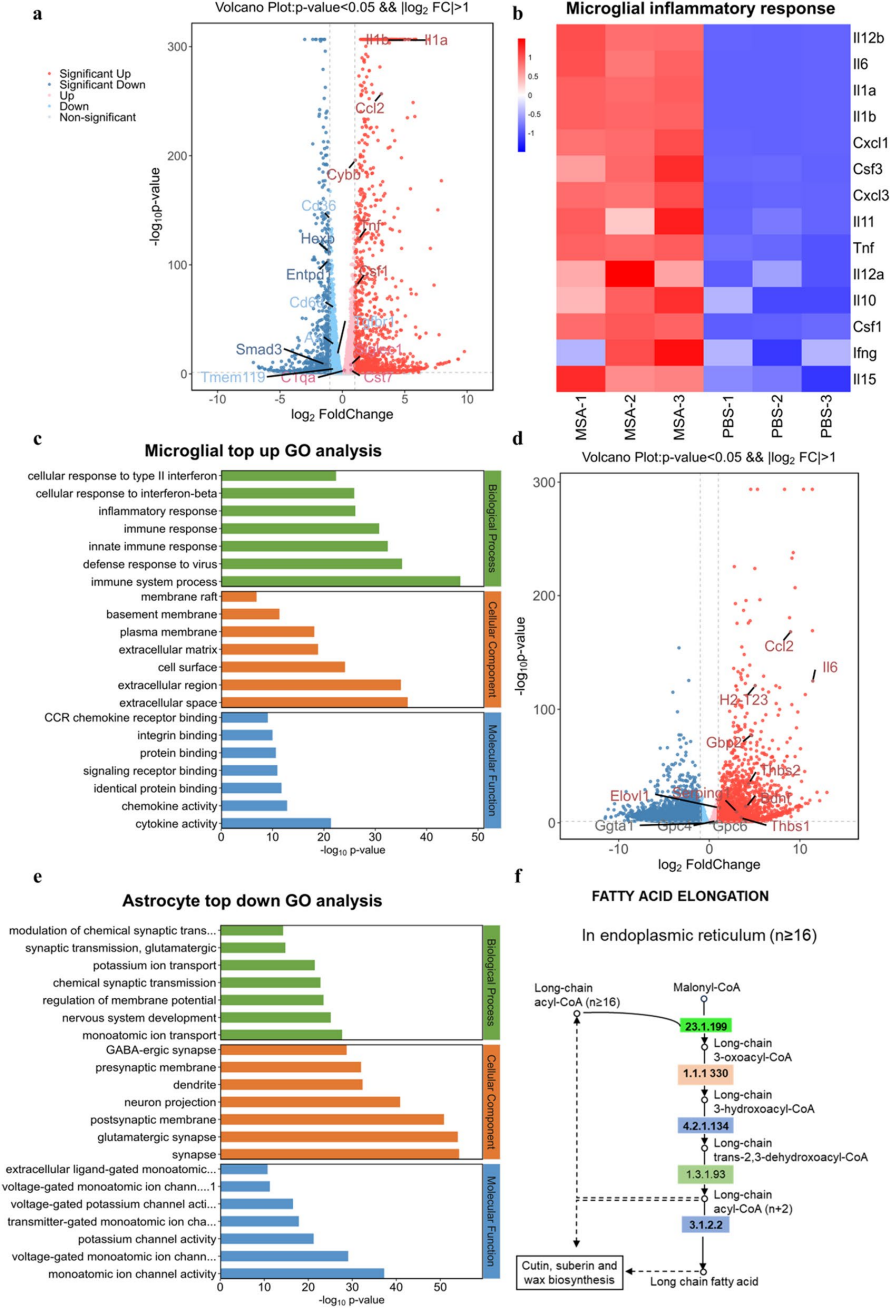
Transcriptome signature of microglia and astrocytes isolated from mice treated with or without MSA. **a** Volcano plot based on differentially expressed genes in the microglia of mice treated with or without MSA. **b** Heatmap showing expression changes in microglial inflammation-related genes in the mice treated with or without MSA. (MSA1-3: microglia isolated from MSA-treated mice, PBS1-3: microglia isolated from PBS-treated mice). **c** GO term analysis showing the most significant upregulated pathway in the microglia of mice treated with or without MSA. **d** Volcano plot based on differentially expressed genes in the astrocytes of mice treated with or without MSA. **e** GO term analysis showing the most significant downregulated pathway in the astrocytes of mice treated with or without MSA. **f** KEGG map showing significantly upregulated fatty acid elongation pathway in the astrocytes of mice treated with or without MSA

In order to further comprehensively explore the effect of MSA on the microglia, astrocyte and neurons in vivo, respectively, we isolated these cells from the mice injected with PBS or MSA, and conducted transcriptome sequencing. The results revealed that in microglia of MSA-treated mice, the transcription of genes associated with M0 (Hexb, Entpd1, Smad3, Tmem119, Tgfbr1) and phagocytosis receptors (Cd36, Cd68, Axl) were significantly decreased, and MGnD-related genes (Ccl2, Cybb, Csf1, Cst7, Siglec1) were upregulated (Fig. 5a). Moreover, Wiki pathway and Gene Ontology (GO) analysis also displayed that MSA substantially activated gene expression related to inflammation in microglia (Fig. 5b, c).

In astrocytes, MSA significantly upregulated neurotoxic astrocyte A1 marker genes (H2-T23, Gbp2, Serping1, Ggta1) and inflammatory factor genes (Ccl2, IL-6). The transcription of genes associated with brain-derived neurotrophic factors (Thbs1, Thbs2, Gpc4, Gpc6, BDNF) were significantly increased in MSA-treated mice (Fig. 5d), which may be attributed to the compensation for the damaged neurons by astrocytes via secreting more neurotrophic factors [42]. Go analysis revealed that MSA infusion down-regulated astrocyte genes related to ion channels, transmembrane transport, and the development of synaptic transmission, which are fundamental for establishing neuronal identity (Fig. 5e). These data suggested that astrocytes in the MSA-treated mouse brains reduced their roles in providing support for neurons. Moreover, KEGG map analysis indicated that astrocytes exhibited remarkable activation in the pathway associated with VLSFAs elongation (Fig. 5f), which significantly increased VLSFAs levels, and was consistent with our in vitro results.

### The effect of MSA on neuron transcriptome

**Fig. 6:**
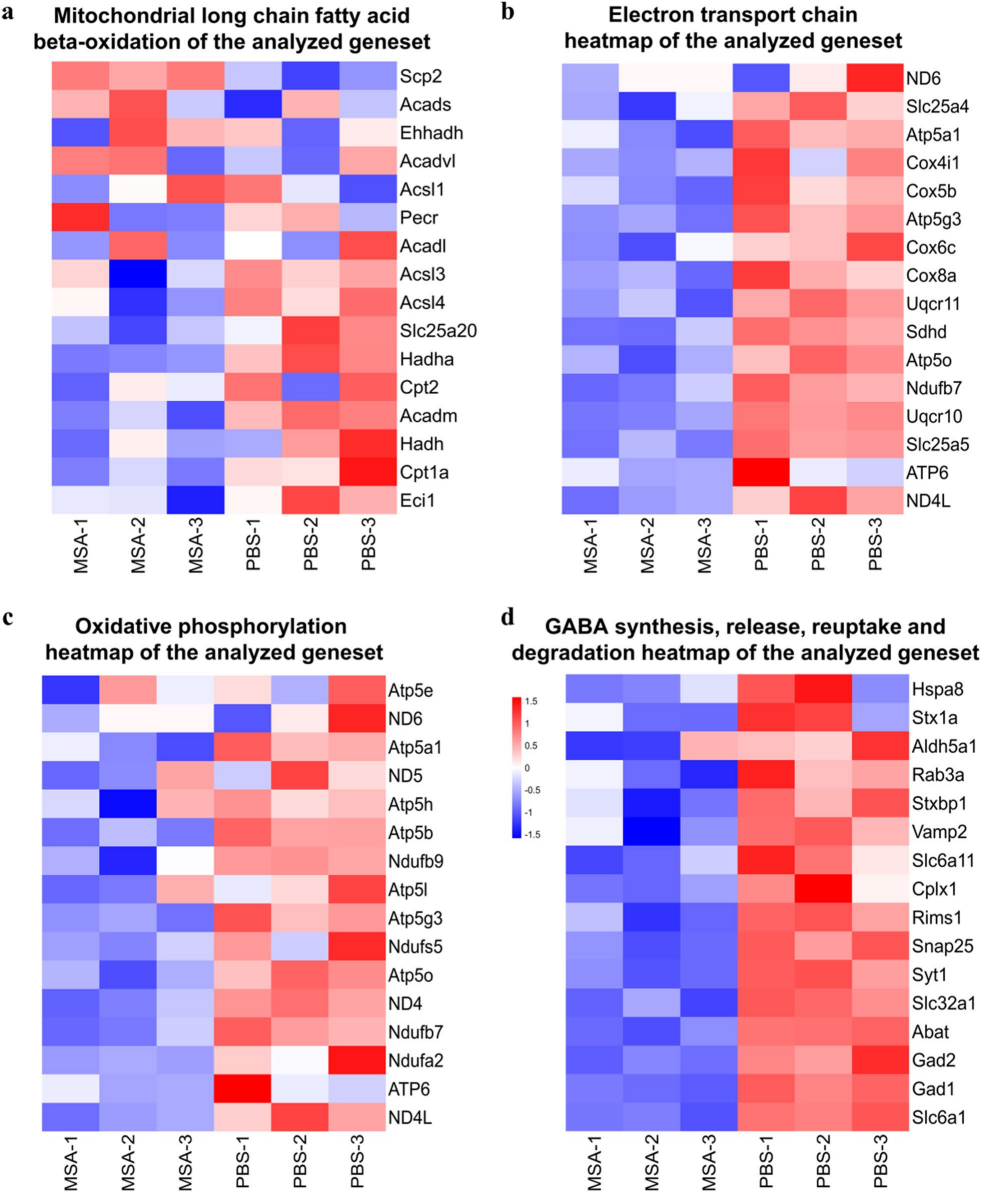
Transcriptome signature of neuron isolated from mice treated with or without MSA. **a-d**, Heatmap showing expression changes of mitochondrial long chain fatty acid beta- oxidation genes (**a**), oxidative phosphorylation genes (**b**), electron transport chain genes (**c**) and GABA synthesis, release, reuptake and degradation genes (**d**) in the neurons isolated from MSA treated mice vs. PBS group mice. (MSA1-3: Neurons isolated from MSA-mice, PBS1-3: Neurons isolated from PBS-mice)

Neurons can take up VLSFAs, while they have very limited capacities for β-oxidation or lipid sequestration for VLSFAs [15], especially for the neurons from MSA-treated mice. Therefore, neuronal mitochondria are directly exposed to the VLSFAs, causing neuronal mitochondrial long chain fatty acid beta-oxidation related genes were significantly decreased (Fig. 6a). Consistently, the heatmaps showed that the expression of electron transport and oxidative phosphorylation in neurons of MSA-treated mice were decreased (Fig. 6b, c). As pre-synaptic mitochondria are necessary to produce ATP to release neurotransmitters, the pathways related to GABA neurotransmitters, including GABA receptor activation and GABA synthesis, release, reuptake and degradation were consequently damaged in the neurons of MSA-treated mice (Fig. 6d), which finally impaired both reference and working/short-term memory. These results indicated that MSA induced microglia and astrocytes to MGnD and A1 phenotypes, respectively, promoting neuroinflammation. A1 astrocytes produced VLSFAs, which impaired neuronal mitochondria function and GABA transmission, leading to decreased the cognition and memory in mice.

## Discussion

Elderly people often present with impaired BBB, and more attention has been paid to the correlation between disrupted BBB and neurodegenerative diseases [27, 28]. Many blood-derived protein such as thrombin, fibrinogen and albumin are prohibited from entering the brain by the intact BBB, but they may enter the brain when BBB is impaired [9, 24]. Albumin, the most abundant protein in plasma, has remained largely unexplored in its role in CNS. Our present study found that IL-1α, TNF-α and C1q secreted by MSA-activated microglia induced astrocytes to their A1 phenotype to generate VLSFAs, the released VLSFAs subsequently triggered neuron apoptosis through PERK endoplasmic reticulum stress response pathway. The injected MSA in mouse brains induced tau phosphorylation via microglial NLRP3 inflammasome pathway, caused neuronal apoptosis, resulting in the decline in learning and spatial memory ability.

Neuron death is one of the most common pathological features in neurodegenerative disorders. Recent evidence showed that VLSFAs played a critical role in neuron apoptosis [12], while VLSFAs generation in astrocytes was dependent on Elovl1. We here found that the levels of VLSFAs and Elovl1 were significantly promoted in astrocytes by C1q, TNF-α and IL-1α in the MSA-induced microglial conditional medium, which was consistent that previous report that neurotoxic reactive astrocytes were induced by activated microglia [23]. Moreover, MSA-induced astrocytic conditional medium failing to induce neuron apoptosis. Thus, MSA-induced neuron apoptosis follows a logic order from microglia to astrocytes to neurons.

Various cells in the brain live in the same complex environment, interact with each other and are subjected to direct or indirect effects of leaked MSA. MSA induced microglia and astrocytes to MGnD and A1 phenotype, respectively, and AM and MM also correspondingly further activated microglia and astrocytes, forming a hazardous microenvironment containing various inflammatory factors, cytokines and VLSFAs in CNS. It was reported that long chain saturated fatty acids promoted inflammation [14], and that there was a positive feedback-loop regulation between VLSFAs and inflammatory factors. We further found that MSA-induced detrimental microenvironment incurred tau phosphorylation and neuron apoptosis, and significantly decreased learning, memory abilities in mice, while Elovl1 knockdown broke this detrimental effect.

Hyperphosphorylated tau, the main component of neurofibrillary tangles in neurons, is a major pathological feature of several neurodegenerative diseases [22]. Hyperphosphorylated tau is apt to aggregate, forming aggregates such as oligomers and fibrils which are toxic to neurons, leading to neuronal death [5]. The present injected MSA in mouse brains induced microgliosis and generation of NLRP3, IL-1β and IL-18. Consistent with previous study [16], these inflammatory factors promoted the generation of tau phosphorylation-related kinases GSK3β and CaMKIIα, leading to obvious tau phosphorylation at multiple sites including Thr181, Ser199, Thr217 and Thr231 (Fig. 3). It has been extensively reported that tau181, tau 217 and tau 231 are desired biomarkers for early diagnosis of AD [25, 26], and brain-derived tau aggregate seeds can spread tauopathy throughout the brain, further inducing the mis-folding of endogenous tau and neuronal degeneration [6, 11]. Therefore, leaked MSA through impaired BBB would be a primary cause for tauopathies. In contrast, MSA did not induce the production of Aβ and α-synuclein, suggesting that MSA mainly contribute to tauopathies rather than other amyloid related pathology.

## Conclusions

In summary, our study here revealed that MSA induced tau phosphorylation and neuron apoptosis based on MSA-activated microglia and astrocytes, respectively, showing the important role of MSA in initiating tauopathies and cognitive decline, and providing a potential therapeutic target for tauopathies.

### Supplementary Information

**Additional file 1. Fig.S1**. MSA activates microglia and astrocytes.** Fig.S2**. MSA does not change neuronal morphology and function.** Fig.S3**. AM activates microglia and induces neuronal excitability.** Fig.S4**. MM activates astrocytes and induces neuronal excitability.** Fig S5**. The effect of inflammatory factors and shElovl1 on Elovl1 expression.** Fig S6**. AAV-shElovl1 effectively knocked down astrocytic Elovl1 in mouse brains.

**Additional file 2. Supplemental Table 1**. List of qPCR primers.

**Additional file 3. Supplemental Table 2**. List of antibodies.

## Data Availability

The datasets used and/or analysed during the current study available from the corresponding author on reasonable request.

## Abbreviations

BBB: Blood–brain barrier
MSA: Mouse serum albumin
VLSFAs: Very long-chain saturated fatty acids
CNS: Central nervous system
CSF: Cerebrospinal fluid
AD: Alzheimer’s disease
FTD: Frontotemporal dementia
GSK-3β: Glycogen synthase kinase-3β
CaMKIIα: Calcium/calmodulin activated protein kinase IIα
Elovl1: Elongation of very-long-chain fatty acid 1
MM: Microglia medium
AM: Astrocyte medium
GM: Glia medium
MAM: Microglia-Astrocyte Medium
AMM: Astrocyte-Microglia Medium
GABA: Gamma-Aminobutyric Acid
NOR: Novel object recognition
MWM: Morris water maze
