# Supplementary material for: Mouse serum albumin induces neuronal apoptosis and tauopathies
Source: Acta Neuropathol Commun. 2024 Apr 23;12:66. doi: 10.1186/s40478-024-01771-6 (PMC11040793; doi:10.1186/s40478-024-01771-6)
Supplement: Supplementary file 1 — Additional file 1. Fig.S1. MSA activates microglia and astrocytes. Fig.S2. MSA does not change neuronal morphology and function. Fig.S3. AM activates microglia and induces neuronal excitability. Fig.S4. MM activates astrocytes and induces neuronal excitability. Fig S5. The effect of inflammatory factors and shElovl1 on Elovl1 expression. Fig S6. AAV-shElovl1 effectively knocked down astrocytic Elovl1 in mouse brains. [file 40478_2024_1771_MOESM1_ESM.docx]

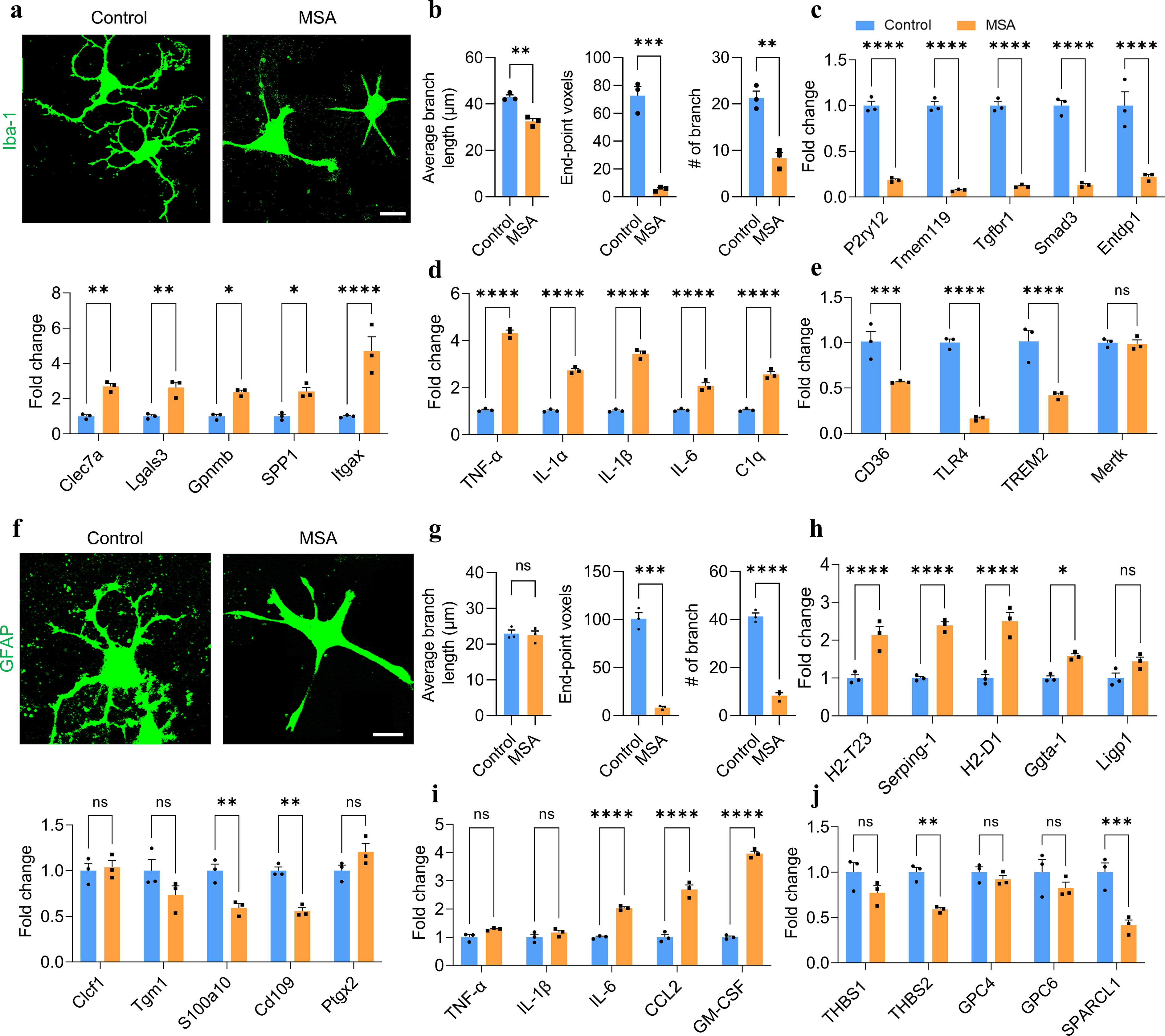


**Fig.S1 MSA activates microglia and astrocytes. a**, Representative confocal images of microglia treated with or without MSA (scale bar, 100 μm). **b**, Statistical analysis of microglial morphology including average branch length, the number of end-point voxels and branch in (a) by Image J. **c**, The mRNA levels of microglial M0 and MGnD markers determined by qPCR. **d**, The mRNA levels of microglial inflammatory factors determined by qPCR. **e**, The mRNA levels of microglial phagocytic receptors determined by qPCR. **f**, Representative confocal images of astrocytes treated with or without MSA (scale bar, 100 μm). **g**, Statistical analysis of astrocytic morphology including average branch length, the number of end-point voxels and branch in (f) by Image J. **h**, The mRNA levels of astrocytic A1 and A2 markers determined by qPCR. **i**, The mRNA levels of astrocytic inflammatory factors determined by qPCR. **j**, The mRNA levels of neurotrophic factors determined by qPCR. For **b**-**e**, **g**-**j**, n = 3, representing three independent experiments, data are mean ± SEM, and Student’s test was used for statistical analysis. ns, no significance; *, *p* < 0.05, **, *p* < 0.01, ***, *p* < 0.001 and ****, *p* < 0.0001.


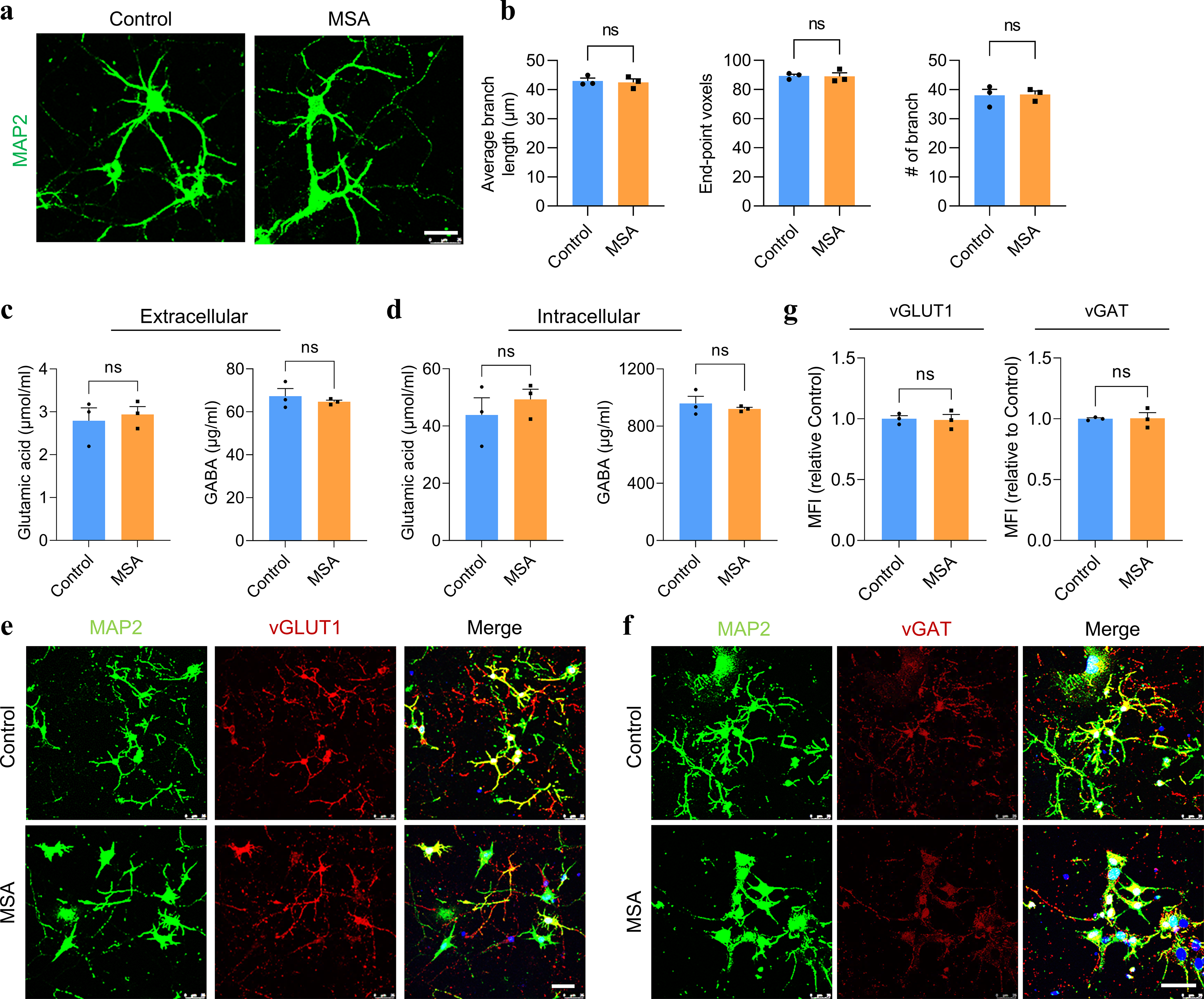


**Fig.S2 MSA does not change neuronal morphology and function.** **a**, Representative confocal images of neurons treated with or without MSA (scale bar, 25 μm). **b**, Statistical analysis of neuronal morphology including average branch length, the number of end-point voxels and branch in (a) by Image J. **c**, The content of neurotransmitter including glutamic acid and GABA in neuronal supernatant. **d**, The content of neurotransmitter including glutamic acid and GABA within the neurons. **e**, Representative confocal images of vGLUT1 in neurons treated with or without MSA (scale bar, 25 μm). **f**, Representative confocal images of vGAT in neurons treated with or without MSA (scale bar, 25 μm). **g**, Statistical analysis the levels of vGLUT1 and vGAT by Image J. For **b**-**d**, **g**, n = 3, representing three independent experiments, data are mean ± SEM, and Student’s test was used for statistical analysis. ns, no significance; *, *p* < 0.05, **, *p* < 0.01, ***, *p* < 0.001and ****, *p* < 0.0001.


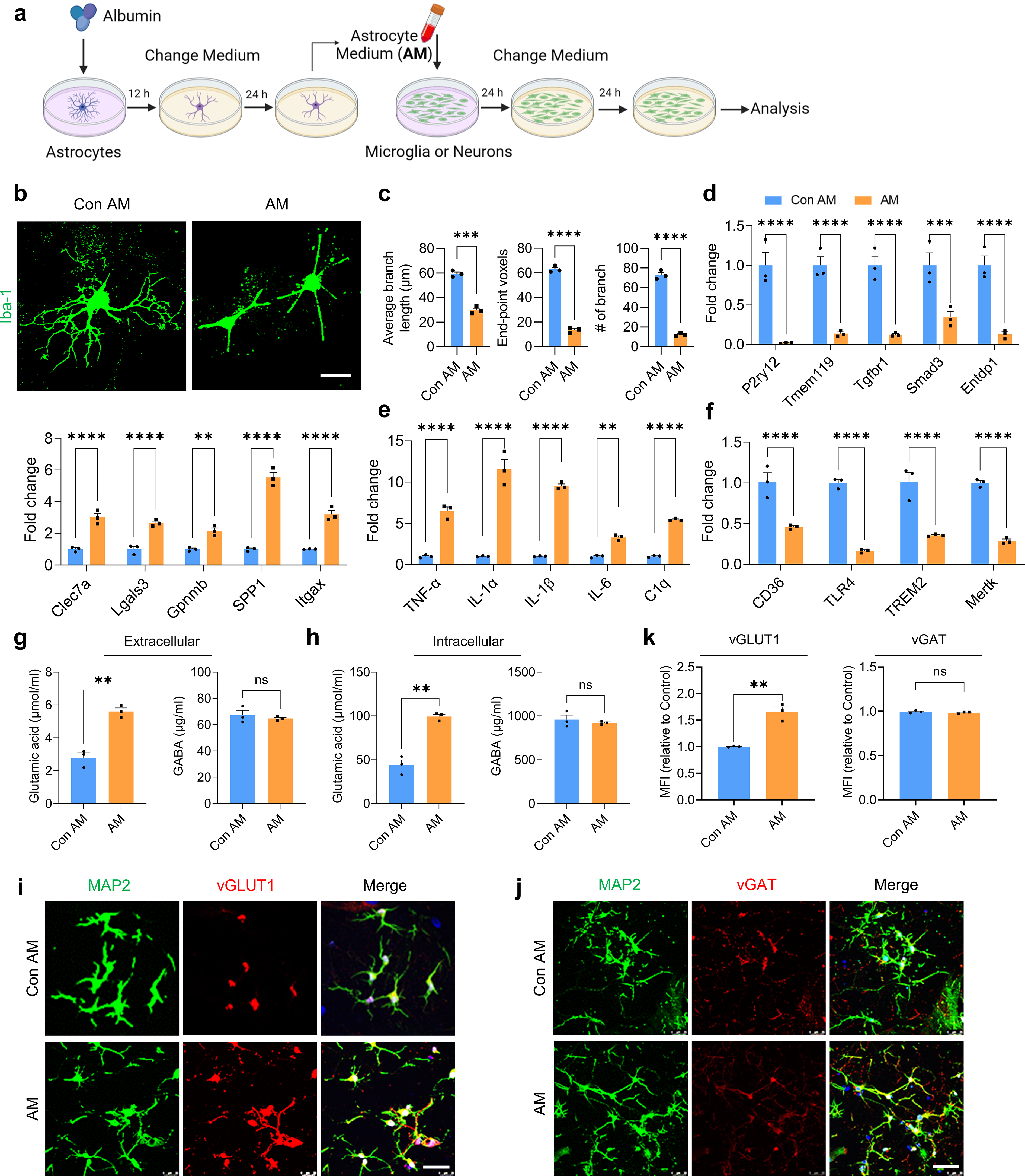


**Fig.S3 AM activates microglia and induces neuronal excitability. a,** A schematic diagram describing the process of microglia and neurons treated with AM, respectively. **b**, Representative confocal images of microglia treated with or without AM (scale bar, 100 μm). **c**, Statistical analysis of microglial morphology including average branch length, the number of end-point voxels and branch in (b) by Image J. **d**, The mRNA levels of microglial M0 and MGnD markers determined by qPCR. **e**, The mRNA levels of microglial inflammatory factors determined by qPCR. **f**, The mRNA levels of microglial phagocytic receptors determined by qPCR. **g**, The content of neurotransmitter including glutamic acid and GABA in neuronal supernatant. **h**, The content of neurotransmitter including glutamic acid and GABA within the neurons. **i**, Representative confocal images of vGLUT1 in neurons (scale bar, 25 μm). **j**, Representative confocal images of vGAT in neurons (scale bar, 25 μm). **k**, Statistical analysis of the levels of vGLUT1 and vGAT in (i) and (j) by Image J. For **c**-**h**, **k**, n = 3, representing three independent experiments, data are mean ± SEM, and Student’s test was used for statistical analysis. ns, no significance; *, *p* < 0.05, **, *p* < 0.01, ***, *p* < 0.001and ****, *p* < 0.0001.


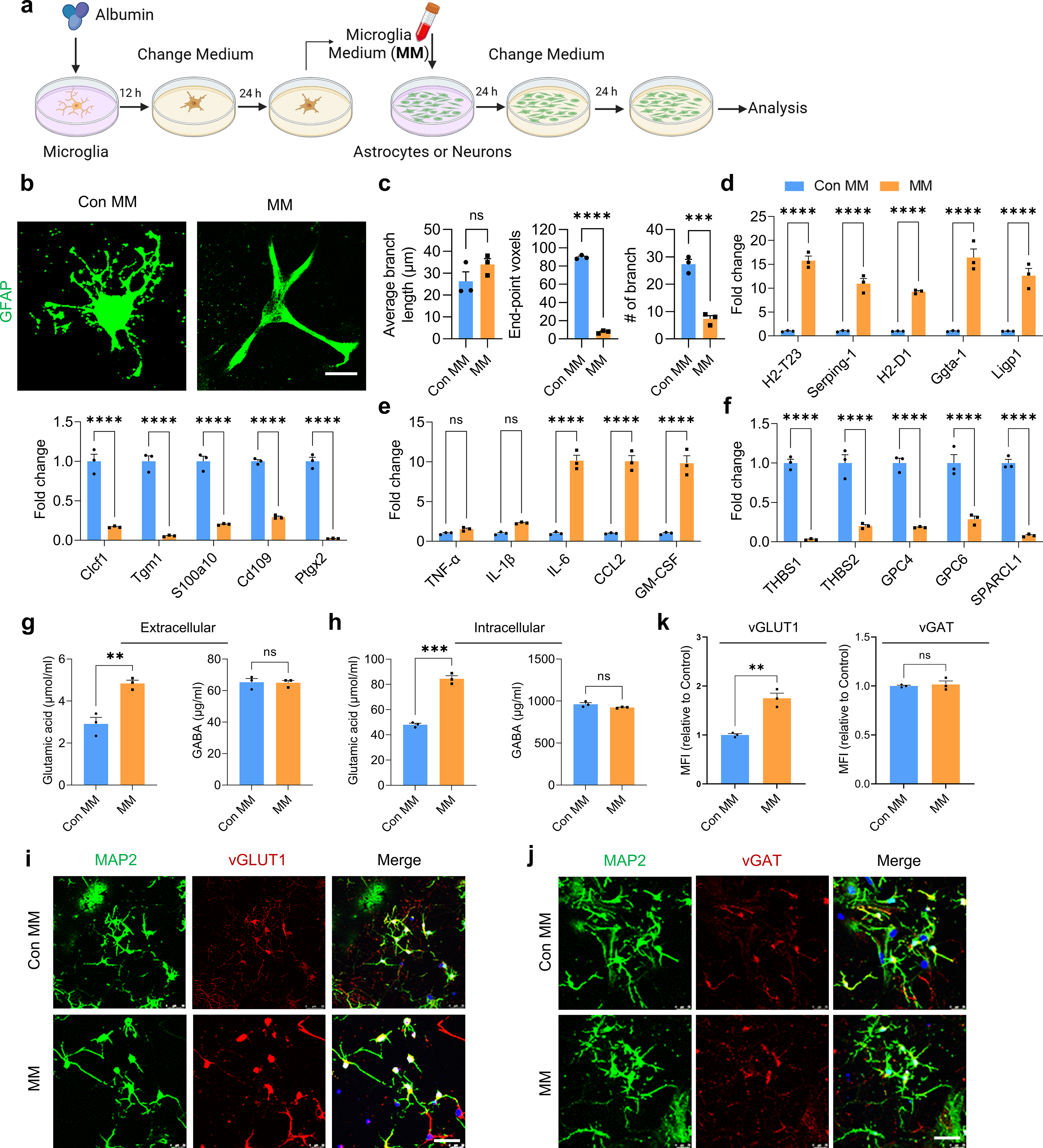


**Fig.S4 MM activates astrocytes and induces neuronal excitability. a,** A schematic diagram describing the process of astrocytes and neurons treated with or without MM, respectively. **b**, Representative confocal images of astrocytes treated with or without MM (scale bar, 100 μm). **c**, Statistical analysis of astrocytes morphology including average branch length, the number of end-point voxels and branch in (b) by Image J. **d**, The mRNA levels of astrocytic A1 and A2 markers determined by qPCR. **e**, The mRNA levels of astrocytic inflammatory factors determined by qPCR. **f**, The mRNA levels of astrocytic neurotrophic factors determined by qPCR. **g**, The content of neurotransmitter including glutamic acid and GABA in neuronal supernatant. **h**, The content of neurotransmitter including glutamic acid and GABA within the neurons. **i**, Representative confocal images of vGLUT1 in neurons (scale bar, 25 μm). **j**, Representative confocal images of vGAT in neurons (scale bar, 25 μm). **k**, Statistical analysis of the levels of vGLUT1 and vGAT in (i) and (j). For **c**-**h**, **k**, n = 3, representing three independent experiments, data are mean ± SEM, and Student’s test was used for statistical analysis. ns, no significance; *, *p* < 0.05, **, *p* < 0.01, ***, *p* < 0.001and ****, *p* < 0.0001.





**Fig S5. The effect of inflammatory factors and shElovl1 on Elovl1 expression. a,** A schematic diagram describing the process of culturing astrocytes with MM while adding neutralizing antibodies or transfecting shElovl1. **b**, Elovl1 mRNA levels in MM-treated astrocytes with or without shElovl1 transfection. **c**, Elovl1 protein levels in MM-treated astrocytes with or without additional shElovl1 transfection. **d**, Quantification of the WB results in (c) by Image J. **e**, Representative confocal images of primary neurons treated with MAM, neutralizing antibodies against IL-1α, TNF-α, and C1q, and shElovl1 transfection (scale bar, 25 μm). **f**-**h**, Statistical analysis of neuronal morphology including average branch length (f), the number of end-point voxels (g) and branch (h)in (e) by Image J. **i**, Representative confocal images of neuronal Tunel^+^ nuclei in red following the treatment with MAM, neutralizing antibodies against IL-1α, TNF-α, and C1q, and shElovl1 transfection (scale bar,25 μm). **j**, The quantitation analysis of Tunel positive cells in (i) by Image J. **k**, The key proteins involved in lipoapoptosis pathway detected by WB. **l**, Quantification of the WB result in (k) via Image J. **m**, mRNA levels of Elovl1 in the astrocytes treated with the various MM neutralized by one, two, or three of anti-inflammatory factor antibodies. For **b**, **d**, **l**, **m**, n = 3, representing three independent experiments, data are mean ± SEM, and Student’s test was used for statistical analysis. **n**, Elovl1 protein levels in MM-treated astrocytes with or without neutralization of three inflammatory factor antibodies. **o**, Quantification of the WB results in (d) by Image J. **p**, Heat map showing the levels of VLSFAs in MAM after different treatment. For **d**, **f**-**h**, **j**, **o**, n = 3, representing three independent experiments, data are mean ± SEM. One-way ANOVA with Tukey’s multiple comparisons test for multiple groups was used for statistical analysis. ns, no significance; *, *p* < 0.05, **, *p* < 0.01, ***, *p* < 0.001 and ****, *p* < 0.0001.


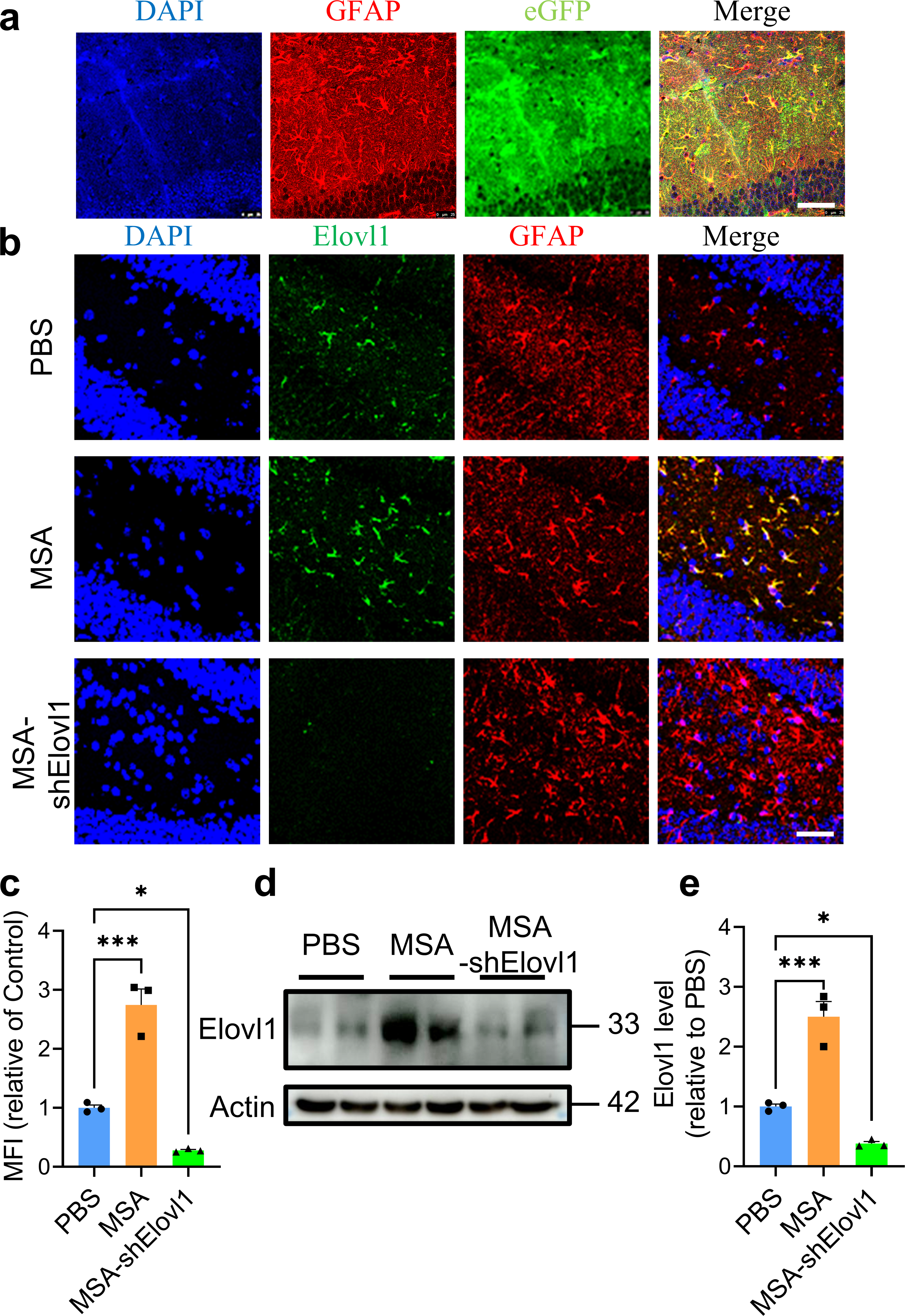


**Figure S6 AAV-shElovl1 effectively knocked down astrocytic Elovl1 in mouse brains. a**, Expression of AAV-shElovl1 in the mouse brains. **b**, Levels of astrocytic Elovl1 in the mouse brains treated with MSA or MSA-shElovl1 detected by IHC. **c**, Quantitative analysis of Elovl1 fluorescence intensity in (b) using Image J. **d**, Total protein levels of Elovl1 in the mouse brains treated with MSA or MSA-shElovl1 detected by WB. **e**, Quantitative analysis of WB bands in (d) using Image J. One-way ANOVA with Tukey’s multiple comparisons test for multiple groups was used for statistical analysis. ns, no significance; *, *p* < 0.05, **, *p* < 0.01, ***, *p* < 0.001 and ****, *p* < 0.0001.

**Supplemental Table 1. Primers used in this study.**

| **Genes** | **Forward primer** | **Reverse primer** | **Size(bp)** |
| --- | --- | --- | --- |
| P2ry12 | CATTGACCGCTACCTGAAGACC | GCCTCCTGTTGGTGAGAATCATG | 91 |
| Tmem119 | ACTACCCATCCTCGTTCCCTGA | TAGCAGCCAGAATGTCAGCCTG | 117 |
| Tgfbr1 | TGCTCCAAACCACAGAGTAGGC | CCCAGAACACTAAGCCCATTGC | 99 |
| Smad3 | GCTTTGAGGCTGTCTACCAGCT | GTGAGGACCTTGACAAGCCACT | 113 |
| Entpd1 | CTGGACAAGAGGAAGGTGCCTA | GACTGTCTGAGATGAGGCTTAGC | 107 |
| Clec7a | CCAGCTAGGTGCTCATCTACTG | CCTTCACTCTGATTGCGGGAAAG | 123 |
| Lgals3 | AACACGAAGCAGGACAATAACTGG | GCAGTAGGTGAGCATCGTTGAC | 98 |
| Gpnmb | GGCTACTTCAGAGCCACCATCA | CTTTGCAGGTCACAGTGAAGTCC | 101 |
| Spp1 | GCTTGGCTTATGGACTGAGGTC | CCTTAGACTCACCGCTCTTCATG | 101 |
| Itgax | TGCCAGGATGACCTTAGTGTCG | CAGAGTGACTGTGGTTCCGTAG | 108 |
| TNF-α | ATGTCTCAGCCTCTTCTCATTC | GCTTGTCACTCGAATTTTGAGA | 179 |
| IL-1β | GCCAGTGAAATGATGGCTTATT | AGGAGCACTTCATCTGTTTAGG | 145 |
| IL-1α | GTTCCTGACTTGTTTGAAGACC | GTTGGACATCTTTGACGTTTCA | 183 |
| IL-6 | CACTGGTCTTTTGGAGTTTGAG | GGACTTTTGTACTCATCTGCAC | 97 |
| C1q | GGACTTTTGTACTCATCTGCAC | GCTTGTCACTCGAATTTTGAGA | 83 |
| CD36 | TTGCGACATGATTAATGGCACAGA | AGATCCGAACACAGCGTAGATAGAC | 118 |
| TLR4 | CGCTCTGGCATCATCTTCATTGTC | CCTCCCATTCCAGGTAGGTGTTTC | 109 |
| TREM2 | ACCGTCACCATCACTCTGAAGAAC | TCCTCCAGCACCTCCACCAG | 119 |
| Mertk | GAAGTTCATGGTGGATATTGCC | CAGTTTCGAGCAGCTAAATCTC | 84 |
| H2-T23 | GGACCGCGAATGACATAGC | GCACCTCAGGGTGACTTCAT | 212 |
| Serping1 | ACAGCCCCCTCTGAATTCTT | GGATGCTCTCCAAGTTGCTC | 299 |
| H2-D1 | TCCGAGATTGTAAAGCGTGAAGA | ACAGGGCAGTGCAGGGATAG | 204 |
| Ggta-1 | GTGAACAGCATGAGGGGTTT | GTTTTGTTGCCTCTGGGTGT | 115 |
| Ligp1 | GGGGCAATAGCTCATTGGTA | ACCTCGAAGACATCCCCTTT | 104 |
| Clcf1 | CTTCAATCCTCCTCGACTGG | TACGTCGGAGTTCAGCTGTG | 176 |
| Tgm1 | CTGTTGGTCCCGTCCCAAA | GGACCTTCCATTGTGCCTGG | 97 |
| S100a10 | CCTCTGGCTGTGGACAAAAT | CTGCTCACAAGAAGCAGTGG | 238 |
| Cd109 | CACAGTCGGGAGCCCTAAAG | GCAGCGATTTCGATGTCCAC | 147 |
| Ptgs2 | GCTGTACAAGCAGTGGCAAA | CCCCAAAGATAGCATCTGGA | 232 |
| CCL2 | TTTTTGTCACCAAGCTCAAGAG | TTCTGATCTCATTTGGTTCCGA | 101 |
| GM-CSF | AGAAGCCCTGAACCTCCTGGATG | CGCCCTTGAGTTTGGTGAAATTGC | 158 |
| THBS1 | GGTAGCTGGAAATGTGGTGCGT | GCACCGATGTTCTCCGTTGTGA | 88 |
| THBS2 | GTATGGAGGGAAGGACTGTGTC | ACTTGGCTCCAGGAAAACACGG | 94 |
| GPC4 | CTGGAGGGTCCTTTCAACATT | GACATCAGTAACCAGTCGGTC | 141 |
| GPC6 | TAGTCCTGTATTGGCAGCCAC | GGCTAATGTCTATAGCAGGGAA | 82 |
| SPARCL1 | GTCACCTGTTTGCTACCAAGTGC | GCCACTTCAAAGTCCGTACAAGC | 129 |
| Elovl1 | GAAGCACTTCGGATGGTTCG | CACCACCAACTCCAGGGAAG | 87 |
| Actin | AAGAGGGATGCTGCCCTTAC | TACGGCCAAATCCGTTCACA | 94 |

**Supplemental Table 2. Antibodies used in this study.**

| Antibodies | Source | Cat. No |
| --- | --- | --- |
| Iba-1 | Abcam | Ab283319 |
| GFAP | Cell Signaling Technology | 3670S |
| MAP2 | Invitrogen | PA1-16751 |
| PERK | Affinity | AF5304 |
| pPERK | Beyotime | AF5902 |
| EIF2A | Beyotime | AG5243 |
| pEIF2A | Affinity | AF3087 |
| FOXO3A | Beyotime | AF609 |
| pFOXO3A | Affinity | AF3020 |
| PUMA | Beyotime | AF0270 |
| BAX | Abcam | Ab32503 |
| Caspase3 | Beyotime | AC030 |
| Cle-Caspase3 | Beyotime | AC033 |
| Actin | Abcam | Ab8266 |
| ELOVL1 | Affinity | AF0670 |
| NLRP3 | Beyotime | AF2155 |
| ASC | Beyotime | AF6234 |
| Caspase1 | Affinity | AF5418 |
| Cle-Caspase1 | Affinity | AF4005 |
| IL-1β | Affinity | AF5103 |
| IL-18 | Beyotime | AF5207 |
| GSK3β | Beyotime | AF1543 |
| CaMKⅡα | Beyotime | AF1639 |
| α-synuclein | Abcam | ab212184 |
| AT8 | Invitrogen | MN1020 |
| TNF-α | Beyotime | AF8208 |
| IL1-α | Abclonal | A22766 |
| C1q | Invitrogen | MA1-40312 |
| vGLUT1 | Abclonal | A12879 |
| vGAT | Proteintech | 14471-1-AP |
| phospho T181 | Abcam | ab254409 |
| phospho S199 | Abcam | ab81268 |
| phospho T217 | Abcam | ab291080 |
| phospho T231 | Abcam | ab151559 |
