# Supplementary material for: Mouse serum albumin induces neuronal apoptosis and tauopathies
Source: Acta Neuropathol Commun. 2024 Apr 23;12:66. doi: 10.1186/s40478-024-01771-6 (PMC11040793; doi:10.1186/s40478-024-01771-6)
Supplement: Supplementary file 2 — Additional file 2. Supplemental Table 1. List of qPCR primers. [file 40478_2024_1771_MOESM2_ESM.docx]

**Supplemental Table 1. Primers used in this study.**

| **Genes** | **Forward primer** | **Reverse primer** | **Size(bp)** |
| --- | --- | --- | --- |
| P2ry12 | CATTGACCGCTACCTGAAGACC | GCCTCCTGTTGGTGAGAATCATG | 91 |
| Tmem119 | ACTACCCATCCTCGTTCCCTGA | TAGCAGCCAGAATGTCAGCCTG | 117 |
| Tgfbr1 | TGCTCCAAACCACAGAGTAGGC | CCCAGAACACTAAGCCCATTGC | 99 |
| Smad3 | GCTTTGAGGCTGTCTACCAGCT | GTGAGGACCTTGACAAGCCACT | 113 |
| Entpd1 | CTGGACAAGAGGAAGGTGCCTA | GACTGTCTGAGATGAGGCTTAGC | 107 |
| Clec7a | CCAGCTAGGTGCTCATCTACTG | CCTTCACTCTGATTGCGGGAAAG | 123 |
| Lgals3 | AACACGAAGCAGGACAATAACTGG | GCAGTAGGTGAGCATCGTTGAC | 98 |
| Gpnmb | GGCTACTTCAGAGCCACCATCA | CTTTGCAGGTCACAGTGAAGTCC | 101 |
| Spp1 | GCTTGGCTTATGGACTGAGGTC | CCTTAGACTCACCGCTCTTCATG | 101 |
| Itgax | TGCCAGGATGACCTTAGTGTCG | CAGAGTGACTGTGGTTCCGTAG | 108 |
| TNF-α | ATGTCTCAGCCTCTTCTCATTC | GCTTGTCACTCGAATTTTGAGA | 179 |
| IL-1β | GCCAGTGAAATGATGGCTTATT | AGGAGCACTTCATCTGTTTAGG | 145 |
| IL-1α | GTTCCTGACTTGTTTGAAGACC | GTTGGACATCTTTGACGTTTCA | 183 |
| IL-6 | CACTGGTCTTTTGGAGTTTGAG | GGACTTTTGTACTCATCTGCAC | 97 |
| C1q | GGACTTTTGTACTCATCTGCAC | GCTTGTCACTCGAATTTTGAGA | 83 |
| CD36 | TTGCGACATGATTAATGGCACAGA | AGATCCGAACACAGCGTAGATAGAC | 118 |
| TLR4 | CGCTCTGGCATCATCTTCATTGTC | CCTCCCATTCCAGGTAGGTGTTTC | 109 |
| TREM2 | ACCGTCACCATCACTCTGAAGAAC | TCCTCCAGCACCTCCACCAG | 119 |
| Mertk | GAAGTTCATGGTGGATATTGCC | CAGTTTCGAGCAGCTAAATCTC | 84 |
| H2-T23 | GGACCGCGAATGACATAGC | GCACCTCAGGGTGACTTCAT | 212 |
| Serping1 | ACAGCCCCCTCTGAATTCTT | GGATGCTCTCCAAGTTGCTC | 299 |
| H2-D1 | TCCGAGATTGTAAAGCGTGAAGA | ACAGGGCAGTGCAGGGATAG | 204 |
| Ggta-1 | GTGAACAGCATGAGGGGTTT | GTTTTGTTGCCTCTGGGTGT | 115 |
| Ligp1 | GGGGCAATAGCTCATTGGTA | ACCTCGAAGACATCCCCTTT | 104 |
| Clcf1 | CTTCAATCCTCCTCGACTGG | TACGTCGGAGTTCAGCTGTG | 176 |
| Tgm1 | CTGTTGGTCCCGTCCCAAA | GGACCTTCCATTGTGCCTGG | 97 |
| S100a10 | CCTCTGGCTGTGGACAAAAT | CTGCTCACAAGAAGCAGTGG | 238 |
| Cd109 | CACAGTCGGGAGCCCTAAAG | GCAGCGATTTCGATGTCCAC | 147 |
| Ptgs2 | GCTGTACAAGCAGTGGCAAA | CCCCAAAGATAGCATCTGGA | 232 |
| CCL2 | TTTTTGTCACCAAGCTCAAGAG | TTCTGATCTCATTTGGTTCCGA | 101 |
| GM-CSF | AGAAGCCCTGAACCTCCTGGATG | CGCCCTTGAGTTTGGTGAAATTGC | 158 |
| THBS1 | GGTAGCTGGAAATGTGGTGCGT | GCACCGATGTTCTCCGTTGTGA | 88 |
| THBS2 | GTATGGAGGGAAGGACTGTGTC | ACTTGGCTCCAGGAAAACACGG | 94 |
| GPC4 | CTGGAGGGTCCTTTCAACATT | GACATCAGTAACCAGTCGGTC | 141 |
| GPC6 | TAGTCCTGTATTGGCAGCCAC | GGCTAATGTCTATAGCAGGGAA | 82 |
| SPARCL1 | GTCACCTGTTTGCTACCAAGTGC | GCCACTTCAAAGTCCGTACAAGC | 129 |
| Elovl1 | GAAGCACTTCGGATGGTTCG | CACCACCAACTCCAGGGAAG | 87 |
| Actin | AAGAGGGATGCTGCCCTTAC | TACGGCCAAATCCGTTCACA | 94 |
