# Supplementary material for: Mouse serum albumin induces neuronal apoptosis and tauopathies
Source: Acta Neuropathol Commun. 2024 Apr 23;12:66. doi: 10.1186/s40478-024-01771-6 (PMC11040793; doi:10.1186/s40478-024-01771-6)
Supplement: Supplementary file 3 — Additional file 3. Supplemental Table 2. List of antibodies. [file 40478_2024_1771_MOESM3_ESM.docx]

**Supplemental Table 2. Antibodies used in this study.**

| Antibodies | Source | Cat. No |
| --- | --- | --- |
| Iba-1 | Abcam | Ab283319 |
| GFAP | Cell Signaling Technology | 3670S |
| MAP2 | Invitrogen | PA1-16751 |
| PERK | Affinity | AF5304 |
| pPERK | Beyotime | AF5902 |
| EIF2A | Beyotime | AG5243 |
| pEIF2A | Affinity | AF3087 |
| FOXO3A | Beyotime | AF609 |
| pFOXO3A | Affinity | AF3020 |
| PUMA | Beyotime | AF0270 |
| BAX | Abcam | Ab32503 |
| Caspase3 | Beyotime | AC030 |
| Cle-Caspase3 | Beyotime | AC033 |
| Actin | Abcam | Ab8266 |
| ELOVL1 | Affinity | AF0670 |
| NLRP3 | Beyotime | AF2155 |
| ASC | Beyotime | AF6234 |
| Caspase1 | Affinity | AF5418 |
| Cle-Caspase1 | Affinity | AF4005 |
| IL-1β | Affinity | AF5103 |
| IL-18 | Beyotime | AF5207 |
| GSK3β | Beyotime | AF1543 |
| CaMKⅡα | Beyotime | AF1639 |
| α-synuclein | Abcam | ab212184 |
| AT8 | Invitrogen | MN1020 |
| TNF-α | Beyotime | AF8208 |
| IL1-α | Abclonal | A22766 |
| C1q | Invitrogen | MA1-40312 |
| vGLUT1 | Abclonal | A12879 |
| vGAT | Proteintech | 14471-1-AP |
| phospho T181 | Abcam | ab254409 |
| phospho S199 | Abcam | ab81268 |
| phospho T217 | Abcam | ab291080 |
| phospho T231 | Abcam | ab151559 |
